# The other blue: Role of sky in the perception of nature

**DOI:** 10.3389/fpsyg.2022.932507

**Published:** 2022-10-28

**Authors:** Izabela Maria Sztuka, Ada Örken, Sonja Sudimac, Simone Kühn

**Affiliations:** ^1^Lise Meitner Group for Environmental Neuroscience, Max Planck Institute for Human Development, Berlin, Germany; ^2^Max Planck Dahlem Campus of Cognition (MPDCC), Max Planck Institute for Human Development, Berlin, Germany; ^3^Max Planck Institute for Human Development, International Max Planck Research School on the Life Course (LIFE), Berlin, Germany; ^4^Department of Psychiatry and Psychotherapy, University Medical Center Hamburg-Eppendorf, Hamburg, Germany; ^5^Max Planck UCL Centre for Computational Psychiatry and Ageing Research Berlin, Germany and London, UK, Berlin, Germany

**Keywords:** sky, greenspaces, environment, nature, GLMM, urban and naturalness

## Abstract

Nature is frequently operationalized as greenery or water to estimate the restorativeness of the environment. Pursuing a deeper understanding of the connection between representation of naturalness and its relationship with restoration, we conducted an experiment aimed to investigate if the sky is perceived as an element of nature. The main goal of this study was to understand how the composition of the environment guides people’s selection of sky as nature in an explicit task. Moreover, we investigated how the amount of visible sky determines this relationship. One hundred five participants participated in a novel explicit judgment task conducted online. In this task, we prepared a set of images trimmed out of 360-degree high dynamic range images. The images were classified according to two primary independent variables representing type of environment (four levels: Nature, Some Nature, Some Urban and Urban) and horizon level (three levels: Low, Medium and High). Each participant was asked to select, by clicking on the image, what they consider as “nature.” In addition, they were asked to judge images on five visual analogue scales: emotional response, aesthetic preference, feeling of familiarity, the openness of the space and naturalness. For analysis, images were segmented into 11 semantic categories (e.g., trees, sky, and water) with each pixel being assigned one semantic label. Our results show that, sky is associated with selections of nature in a specific pattern. The relationship is dependent on the particular set of conditions that are present in the environment (i.e., weather, season of the year) rather than the type of the environment (urban, nature). The availability of sky on the image affects the selection of other nature labels with selections more likely when only a small amount of sky was available. Furthermore, we found that the amount of sky had a significant positive association with the naturalness rating of the whole image, but the effect was small. Our results also indicate that subjective selections of sky predict the naturalness better than trees and water. On the other hand, objective presence of trees and water has a stronger positive association with naturalness while objective presence of sky is positively associated with naturalness. The results show that, relative to its availability sky is considered as nature.

## Introduction

Within the process of global urbanization, the population is moving away from natural environments into man-made urban centers (Mac Kerron and Mourato, 2013; Pelgrims et al., 2021; Mouratidis, 2022). In the coming decades, the population will continue to concentrate in urban areas (Ritchie and Roser, 2018). That shift in habitats stimulates growing academic interest in its implications for population health. A major line of research is the identification of the salutogenic factors of living environments for the purpose of informing future policymaking and supporting global population wellbeing.

Part of the discipline of environmental psychology is focused on unravelling the core principles of the beneficial effects of nature on psychological wellbeing. The theoretical narrative has built its assumptions around the principle of the restoration of depleted adaptive resources. Two theories about processes through which people benefit from non-threatening nature underlie most of the current evidence. (1) Stress reduction theory (SRT) (Ulrich et al., 1991) assumes that access to nature benefits emotional restoration and helps offset the effects of stress. (2) Attention restoration theory (ART) (Kaplan and Kaplan, 1989) posits that natural environments aid in recovery from mental fatigue, in particular, attentional capacity. Since then, an extensive body of evidence has shown different effects of nature on the mind and brain. Currently, evidence suggests the beneficial effects of nature on improved attentional capacities (Berman et al., 2012), stress recovery (Valtchanov and Ellard, 2015), anxiety, and depression (Kotera et al., 2021). Recently, these investigations extended into the neuroscientific domain (Bratman et al., 2015; Kühn et al., 2017). Research on the effect of a casual walk in the forest on the amygdala, which has been linked to stress processing (Sudimac et al., 2022), indicates that amygdala activation decreases after a walk in nature, whereas it remains stable after a walk in an urban environment. Thereby the result suggests an explanation for the salutogenic effect of nature on brain functional physiology.

Green and blue spaces have long been investigated as sources of beneficial effects of nature (White et al., 2021). Green spaces refer primarily to parks, forests and other predominantly vegetated areas. So-called “blue spaces” traditionally refer to water bodies: rivers, ponds, lakes etc. Such interpretation resulted in research prevalently measuring the effect of nature as conveyed by these studied components (e.g., trees, lakes). The study by Berto (2005) used pictures of green and blue spaces (trees and water) and found that exposure to these restorative features would improve attentional capacity. In the experiment by Berman et al. (2008), participants were sent on a predefined, one-hour walk in an arboretum with a path specified within green spaces. The effect on attention restoration was compared to the effect of a walk on a busy urban road. The arboretum walk was found to substantially improve working memory performance. In a larger-scale study (Dadvand et al., 2015), green space in the direct neighborhood was associated with cognitive development among schoolchildren.

To better account for global environmental diversity, the research has recently more extensively explored the “blue” surface: namely water. In an interesting review of the cross-disciplinary evidence on the subject, White et al. (2020) outlined the notable differences between green and blue space effects on health and wellbeing outcomes. The reviewed evidence revealed a wide array of relationships between diverse interactions with blue spaces and lower risks to mental health. What has been rather in the background of scientific focus so far is that nature has one more significant “blue”: the sky.

The sky is a space and surface that lies above the horizon and the surface of the Earth. The sky’s physical properties have a contextual impact on how the environment is perceived. An example of such an effect is the influence of the spectral frequency of light and color and change the physical conditions and visual properties of the environment (sunshine, rain, snow, and sand). Spatially, sky delimits the view on the horizon forming the skyline, aiding in shaping the perception of scenes’ depth and openness of the view. Visually, sky generates a sense of a physical barrier, encircling the environment from each of the three-dimensional directions. Therefore, it is reasonable to assume the sky has a significant potential to influence the environment (Li and Du, 2021). Consequently, it holds the capacity of arbitrating the effects of nature on mental health. Since nature and sky exposure are frequently concurrent, Beute and de Kort (2013) pursued the investigation of the parallel effect of naturalness and daylight to find that there is explicit preference for bright, sunny, and natural scenes. The assumption of interaction between sky, view and environment is at the core of the recent extensive review by Beute (2022) who gathered compelling evidence of beneficial characteristics of window views, connecting the properties of daylight, views, and windows. In the review, several studies referred to the latent effect of sky and its condition. They indicated mixed outcome on the measured effect (notably preferences and restorativeness) with some evidence indicating no effect of sky on wellbeing or higher satisfaction with the views. In these studies, sky is conceptualized as an integral part of the window views and hence investigated as part of the overall effect. The review points out that frequently used outcome measures assess subjective wellbeing, preference and broadly defined restorativeness. In the 2018 experiment Masoudinejad and Hartig (2018) investigated how much sky and other components of the scenes in images affect expectations that urban window view will have a restorative effect. The result showed that views with increased visibility of sky were judged as the most restorative. Later, in a virtual reality study of window view effect Masoudinejad and Hartig (2018) showed that visibility of the sky was positively related to emotional restorativeness. Interestingly, no effect of weather type was found. An openness of the space study by Raanaas et al. (2011) showed that an unobstructed window view of natural surroundings supports clinical rehabilitation. Using a different approach of direct exposure rather than window view Asgarzadeh et al. (2014) showed that sky visibility improves the perception of spaciousness. In their investigation of the relationship between gaze patterns and view preference Batool et al. (2021) observed that while verbally describing the preferred view, participants indicated importance of sky and patterns visible on it to their preference scene.

Further strengthening the importance of including the sky in environmental psychology research comes from work on seasonal affective disorder (SAD). SAD is now clinically classified as a major depressive disorder (Diagnostic and statistical manual of mental disorders: DSM-5™, 2013). The disorder is widely associated with reduced availability of natural light during the winter months and decreased outdoor activity, a phenomenon that could likewise be connected to sky exposure (Sarran et al., 2017; Fonte and Coutinho, 2021). However, overall “the other blue” – the sky has attracted little direct attention in comparison to vegetation and water.

The evidence so far arbitrarily assumed that sky is an expression of naturalness as a part of visual world. To what extent people actually perceive the sky as part of nature is unknown. This gap in the empirical evidence concerning the sky seems to be substantial but depends on a significant factor. So far, the intention of experimental methodologies is to assemble the stimulus set (as images, videos, or walks) that expresses the naturalness of the environment through the perspective of particular landscape characteristics. As a result experiments investigate the effect through the lens of specific landscape features, i.e., tree density, coverage of green spaces, or water bodies. Berto (2005) explicitly mentions the representation of trees or sea as guiding the image selection. In their study of the influence of naturalness on expressed thoughts, Schertz and Berman (2019) assembled park pictures that convey specific ground layout patterns aimed at capturing involuntary attention through soft fascination. While investigating the effect of walking in nature on neural activity in the prefrontal cortex, Bratman et al. (2015) designed the walk in nature based on an understanding of the natural environment as an urban greenspace composed of grassland, woodland, and wide ocean view. The studies discussed by Beute (2022) that indeed incorporated sky into designs, did so while considering sky as a secondary component separate from landscape in the overall window view or scene. In essence, sky is neither considered “green” nor “blue.” Yet, the effect is most likely latently present in the measured outcomes. There is considerable scholarship showing the positive influence of viewing images of nature with the effect driven in a bottom-up manner by effects of low-level image statistics (Berman et al., 2014; Kardan et al., 2015; Ibarra et al., 2017). However, these studies approach the view of a scene as entirely natural or urban, globalizing the effect of image statistics, without further separating the context. The interaction between the low-and high-level visual processing is well-evidenced to be highly interactive (Groen et al., 2017). Hence the assumption of combined effect of image statistics and category content on how people perceive nature is most likely. However, none of the studies in environmental psychology so far explicitly investigated it. In this paper, we focus on bringing the sky into the framework of nature perception while investigating the content context inside the scenes. Potential interactions with low-level visual features falls outside of scope of this paper.

Our objective is to begin to close the above-mentioned gap in environmental psychological research by explicitly investigating the role of the sky in the perception of nature. We aim to overcome the empirical ambiguity in the research by directly studying the effect of the perceived amount of sky and its conditions. In the current paper, we present our investigation showing that the presence of sky in diverse environmental contexts and different amounts impacts participants’ choices on what they would refer to as “nature.” We expect these decisions to affect their judgment of naturalness. For our research, we defined naturalness as formed by the properties of the environment, scene or object, which are either present or mimic the properties of the organic world. To operationalize our assumption we designed a novel paradigm in which we combined a free-selection task with the subjective judgment of images containing outdoor scenes. Our methodology allowed participants to explicitly select what they considered “nature” on images. The scenes represent types of environment, varying levels of horizon, weather conditions and seasons of the year. Unlike the previous research, analyzing the sky as a component of window view (Beute and de Kort, 2013), which frequently focused on categorizing environments as urban or nature, our design accounts for diversity of circumstances where these two categories overlap and interact. Such an approach follows the suggestion of Hartig (2021) who argues that from a social and behavioural point of view, humans in cities perpetuate the same natural processes as in other habitats. In order to more accurately control for the amount of sky and sky conditions we have manipulated the horizon level in the scenes and accounted for common weather patterns affecting the view. We measured how frequently the sky was chosen as an element of nature, in what quantities and context. The resulting nature-selections were expressed in quantitative (i.e., number of selections, sky selections) and qualitative measures (semantic vector of features selected). Moreover, for each image, we also collected subjective judgments of aesthetic preference, feeling of openness of the space, perceived naturalness, and invoked emotional response (stress-relaxation dimension). Consequently, the study models the direct relationship between sky, environment, and individuals’ internal representation of nature in distinct contexts.

## Materials and methods

### Participants

The study was approved by the Local Psychological Ethics Committee at the Center for Psychosocial Medicine (LPEK-0280) at University Medical Center Hamburg-Eppendorf, Germany. Our study recruited a sample of 113 healthy participants *via* the experimental platform Prolific in the United Kingdom. The size of required sample was established by power calculation performed in WebPower (Zhang and Yuan, 2018) with expected medium effect size of 0.4. The data obtained in the study were carefully inspected for irregularities. Data from two participants were excluded due to failure to complete the task. Additionally, we excluded six participants because their answers to the socioeconomic status questionnaire were not recorded due to a technical error. In total, eight participants were excluded from further analysis. Out of 105 participants included in the final analysis 51 were females (*M*_age_ = 27.6, *SD*_age_ = 7.38) and 54 were males (*M*_age_ = 27.5, *SD*_age_ = 8.08). All participants were required to have access to a screen size between 13″ and 20″. The inclusion criteria also included the absence of a history of neurological and psychological disorders and traumatic brain injury. Data collection was performed online in July 2021 in the United Kingdom, during the coronavirus pandemic, however outside of the period of extensive and prolonged country-wide lockdown. Nevertheless, the authors would like to highlight that the outcome of the study may be affected by these circumstances affecting the participants responses. Out of 113 participants 49 declared that they were currently working from home and declared that they have been working from home for an average of 12 months (*SD*_WFH_ = 7). Due to the pandemic restricting the movement we have also collected data on the associated time spent outdoors. Participants working from home spend daily on average 2 h 30 min outdoors (*SD*_outdoor_ = 1.45) while participants not working from home spend on average 4 h 15 min outdoors (*SD*_outdoor_ = 2.90).

### Stimuli

The images used in the experiment were created from selected 48 High Dynamic Range Images (HDRI)—a 360-degree hemispherical images sourced from the HDRI database HDRIheaven.^1^ Each HDRI had to fulfil the following criteria: no visible people in the foreground, no landmarks, visible sky, minimal banners or text and minimum 8 k quality. The HDRIs were chosen from a database using a metasearch engine with keywords: “nature” and “urban.” Three independent evaluators classified the selected images into four categories: urban, some urban, some nature and nature. These categories reflect the levels of primary independent variable. Each category contains an equal number of 12 HDRI environments. We defined environmental categories as follows:

- (U)Urban: A landscape strongly dominated by the presence of man-made structures with few to no natural elements.
- (SU)Some urban: A landscape with mostly man-made structures but including some natural elements.
- (SN)Some nature: A landscape where natural elements have a significant presence, including some man-made structures.
- (N)Nature: A landscape with a complete absence of human alterations and structures.

**Figure 1:**
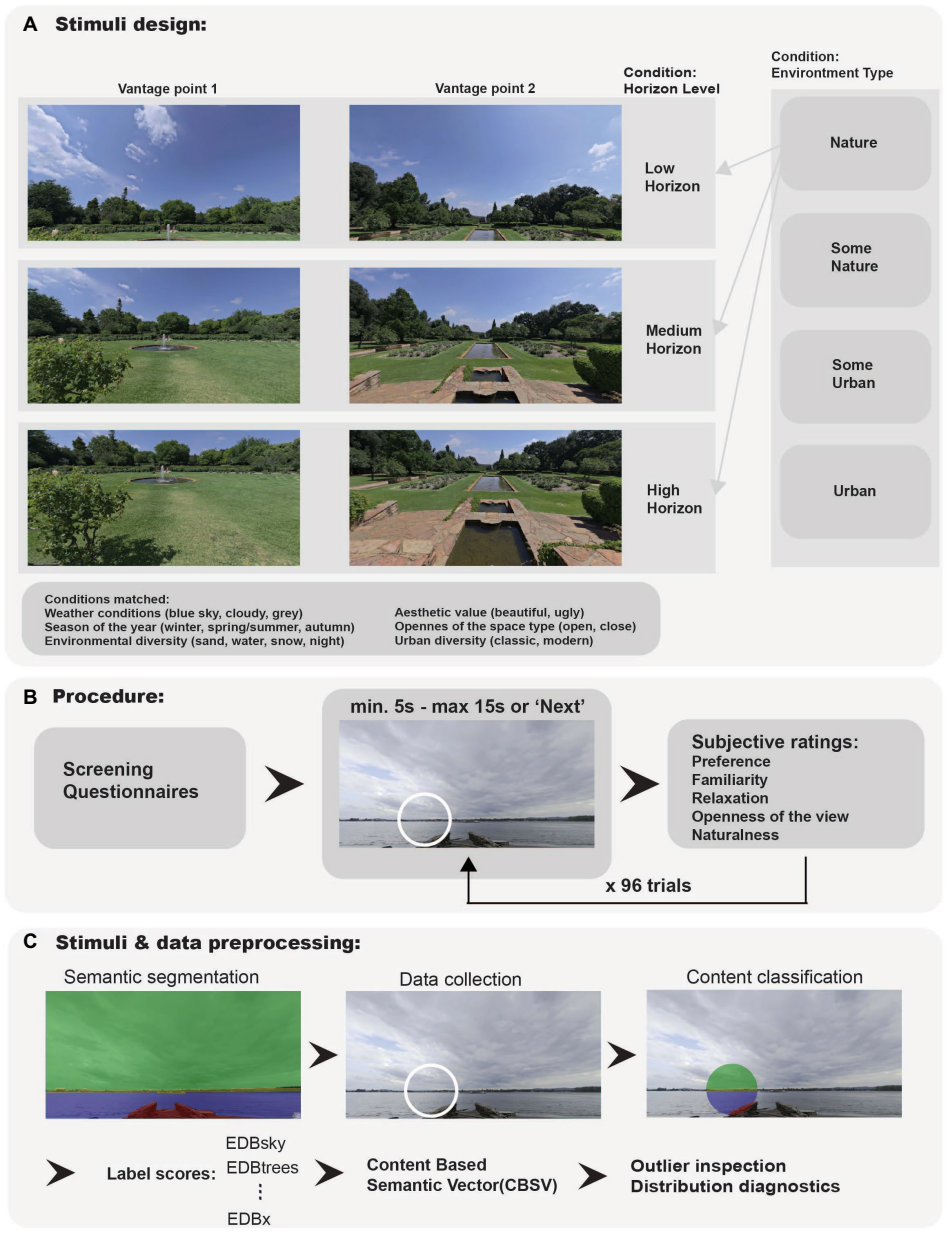
**(A)** Stimuli design. **(B)** Procedure. **(C)** Stimuli and data pre-processing.

Additionally, the image set accounted for covariates of diverse weather conditions and seasons of the year. Finally, out of each HDRI environment, 6 image trims were taken using Adobe Photoshop 2021 3D Spherical Panorama tool^2^ (see Figure 1A for an example). Six trims corresponded with the following 2 vantage point conditions creating two image triplets per environment: front image (0-degree camera rotation), back image (roughly 180-degree camera rotation); and 3 horizon location conditions: low horizon, medium horizon and high horizon. These conditions correspond with the level at which the horizon was present on the trimmed image. Control of vantage point was added during the stimulus set preparation to assure the control for the within-HDRI environment variance of the view. As a result of trimming, a total of 288 images were created and resized to 1500 × 750 pixels with 72 pixels/inch of resolution.

Following trimming, all 288 images were then semantically segmented into the following categories of interest: trees, sky, grass, water, rocks, sand, vegetation, snow, manmade, and background (used for unclassified or blurred pixels). Categories were found to be most common occurring to the environments included in the experimental image set. The segmentation was performed using LabelMe, an open-source annotation tool (Wada, 2016). The resulting annotation matrix was converted into a semantic vector by quantification of the surface of each semantic label on the image (i.e., the percentage of the surface taken by sky on the image). Such an approach allowed us to identify the semantic vector of choices made by participants. Additionally, the final image set was annotated with a categorical vector representing weather conditions in the sky: clear, grey, and cloudy; and seasons: winter, autumn, spring, and summer.

As the last step, based on semantic segmentation of the images we have created additional covariates: percentage of the sky on the image, percentage of nature (quantified percentage of surface covered by semantic labels associated with natural environments), total percentage of greenery (grass + trees + vegetation).

### Design

In order to obtain the subjective evaluation of the images, we conducted a 4 × 3 × 2 factorial mixed-design experiment that used a randomized subset of 96 images (out of 288). The manipulated independent variables within the image set are the type of environment (U,SU,SN,N – levels described above), horizon level (Low, Medium and High) and vantage point (front, back) within the original HDRI environment. The key variables are described in detail in the previous section (Stimuli). However, each participant saw only one horizon condition per image representing each vantage point (see Figure 1A). The variance in horizon condition was introduced to ensure that participants will remain unaware of the experimental manipulation and to increase the paradigms’ internal validity. In consequence, the presented image set differed for each participant. Therefore, in the analysis, we introduced participant and image as having a random effect on the outcome.

### Subjective rating scales

As a part of the experimental procedure, we have asked participants to rate the images on five subjective scales. Each scale assessed a different aspect of response evoked by the image. First, we asked for aesthetic preference dislike – like commonly used in experimental designs investigating environmental effect. We also asked how familiar the scene was to the participant unfamiliar – familiar. We asked participants to rate the feeling of emotional response to the image—whether it made them feel more stressed or relaxed. The scale followed the theoretical assumptions of ART assuming the natural environments are found more restorative. Furthermore, we asked participants to subjectively judge the openness of the view (closed view – open view). Finally, we have asked participants to judge naturalness of the scene. With the exception of naturalness, all ratings were judged on the scale from −50 to 50 with default set of the cursor on 0. In the case of naturalness, we have asked participants to judge it on the scale 0 to 100, with 0 indicating no naturalness and 100 indicating full naturalness. Contrary to insofar approaches, we chose not to use artificial-natural binomial scale. We chose not to focus participants on assessment of manmade elements of the scene but instead asked them to assess the quality of its natural characteristics forming the perception of naturalness.

### Questionnaires

As a part of the project, we have also collected a set of covariates in the form of well-established scales and questionnaires as well as a set of information about participant’s environmental living conditions. A full list of collected items is available in the Supplementary material: List of covariates. Apart from standard demographical questions, we have used a personality questionnaire 10 item big five questionnaire (BFI-10; Rammstedt and John, 2007), a state and trait anxiety inventory (STAI; Spielberger et al., 1983), aesthetic responsiveness assessment scale (AREA; Schlotz et al., 2020), and connectedness to nature scale (CNS; Mayer and Frantz, 2004). As a part of the questionnaire, we have also asked questions living conditions during upbringing [based on urbanicity score by Lederbogen et al. (2011)], current living conditions, whether they work from home at the moment and if so, for how long. Moreover, we have asked participants to name three words that came to their mind, associated with the word “nature” and “urban.” These covariates are not the focus of this particular analysis. Therefore, they are not part of the paper.

### Procedure

The experiment was delivered online using the Prolific testing platform with a paradigm programmed in Java Script (Czienskowski, 2021). After the initial screening for exclusion criteria, participants were first asked to answer questions about their socio-economic status, living conditions, STAI, BFI-10, AREA, and CNS questionnaires. Afterward, in a novel free-selection task participants were presented with each of the 48 environments from two different vantage points, answering 96 randomized image trials in total. Each participant saw 8 images from each of the 12 conditions (3 horizon level × 4 nature category) in randomized order (Figure 1B).

For each image trial, the task was to select areas on the images that participants considered nature. The selection was made with a circle frame of a 150-pixel radius that was operated with a computer mouse or touchpad. Participants had 15 s to answer and an unlimited number of selections. However, they could not move on to the next image during the first 5 s. After 15 s or when participants clicked on the “Next” button, the application proceeded to the evaluation. Here participants saw a smaller version of the image with scales depicted below and were asked to subjectively evaluate the image based on five scales: familiarity, naturalness, the openness of the view, emotional response (feeling relaxed – stressed), and preference (Figure 1B). The trial sequence was repeated 94 times.

### Data preprocessing

Preprocessing was performed in MATLAB 2020b (The MathWorks, Inc., Natick, Massachusetts, United States). Task-related data were preprocessed in two steps. First, we summed the number of clicks made by each participant on each image. These were inspected for outliers. All values above 20 were removed as well as indicated extreme behaviors (rapid, serial clicking in one area) that were not in line with the task. By extension, we investigated if these occurrences had patterns across specific subjects or images. Nevertheless, none of the participants or images was identified to cause the significant anomalies. Afterward, the selections made by participants were individually preprocessed using a content-based semantic logical classifier which compared the semantic content of each selection per click, image and participant with a previously semantically annotated file of the image (Figure 1C). The classifier calculated the content-based semantic vector (CBSV), which contained scores of relative total label selected (EDB) on the image. For each image, participant, click and semantic label the following score was computed:


$$Score\;EDB_{x}=\sum_{n}\left(\frac{D_{x}}{B_{x}}\right)\times 100$$

where, *n* is the number of the respective click, *x* a label, *Dx* total of pixels classified as label *x* within the selection circle and *Bx* is the total of pixels classified as label *x* in the image. Overall, the score is the expression of how much in total label *x* was selected by a participant on the image. Following the computation of EDB scores, the CBSV was inspected in a data quality check. We followed Zuur et al. (2010) description of the data preprocessing procedure. In CBSV, outliers across the labels expressed extreme behaviors. Such activity was detected in vectors of sky and rocks where participants’ EDB indicated that they significantly exceeded the available surface of the label (serially clicking in the same spot). The activity was limited to specific participants and images. Hence, we decided to remove only extreme activity or the entire image rather than the participant. As a result, the total number of images decreased to 258. In the case of the sky vector, we removed all values over 100 and absolute zeros. The latter resulted from experimental design. The cases where no selection was made resulted from the absence of sky (or very minimal presence <2%). It was the only vector where such elimination was performed due to its importance for the research question. For the resulting dataset (*n* = 6,397), we calculated the descriptive statistics for the semantic labels (see Supplementary material: Selection summary statistics).

In the case of subjective ratings, data diagnostic did not yield any outliers (Leys et al., 2013). However, there were many answers at the extreme points of the scales (for example, distribution, see Supplementary material: Distribution plots). We did not remove any of the extreme answers, since they were not statistical outliers. It seems that some participants may have treated the scales as binomial yes/no questions, although they were continuous.

Lastly, we have calculated the outcomes of covariate questionnaires: CNS, BFI, STAI, and urbanicity scores. However, these covariates are not the part of the analysis.

### Data analysis

All statistical analyses were performed using R Statistical Software (v 4.1.2; R Core Team, 2022) and Python programming language (v 3.9; Python Software Foundation^3^). The list of packages necessary to reproduce the analysis is available in the Supplementary material: Package list.

The Kolmogorov–Smirnov (K-S) normality tests indicated that data did not conform to a normal distribution (*p* < 0.001) across the essential label sky and subjective ratings (Supplementary material: Distribution plots). Visual inspection revealed that our dependent variables were skewed: sky toward either lower values (selection scores) or binomially (ratings). Moreover, Levene’s test indicated also a violation of homogeneity of variance (*p* > 0.001) across our experimental factors. Notably, both tests have decreased sensitivity to sample sizes bigger than 5,000. Consequently, we used robust methods to predict the effects of our experimental covariates.

Due to data normality violations, we have applied a non-parametric method to test the main free-selection task assumptions. The main study hypotheses required us to apply robust statistical modelling. Generalized linear mixed-effects models (GLMM) provided a solution to the violation of normality assumption across outcome variables, without the necessity to overly transform variables. GLMM enabled us to meet the mathematical criterion of normalized homoscedastic residuals in linear modelling. Transformation might have normalized the residuals but also distorted the ratio scale of measured variables. The GLMM approach allowed for differences between individuals to be properly assessed using metric context. Moreover, GLMM is capable of accounting for random populations that share nested relationships such as our experimental design. In GLMM analysis, we investigated the relationships between the subjective ratings and the amount of sky present in the images as well as the selection of sky as nature. In order to test if the design has the same impact on tree selection scores, we fitted an additional model with the obtained EDB scores of trees selection. Finally, we fitted the naturalness rating with GLMM models of tree and water selections with a random effect of the subject and image. A detailed description of GLMM computation is available in Supplementary material: GLMM computation.

## Results

### Paradigm validation

To ensure our horizon categorization reflected the visibility of the sky, we checked if there were statistically significant differences between horizon level categories (Low, Middle and High) in the quantified percentage of sky coverage.

The one-way ANOVA indicated a statistically significant effect [*F*(2,158.6) = 142.34, *p* < 0.001 η^2^ = 0.500] of the horizon level on the amount of sky in the images. Low horizon condition images on average contained 41.41% (*SD* = 23.02) of the sky surface, Medium 18.85% (*SD* = 13.1) and High 3.91% (*SD* = 4.42). *Post hoc* multiple comparison test with Bonferroni correction indicated significant differences (*p* < 0.001) between all levels of condition.

**Figure 2:**
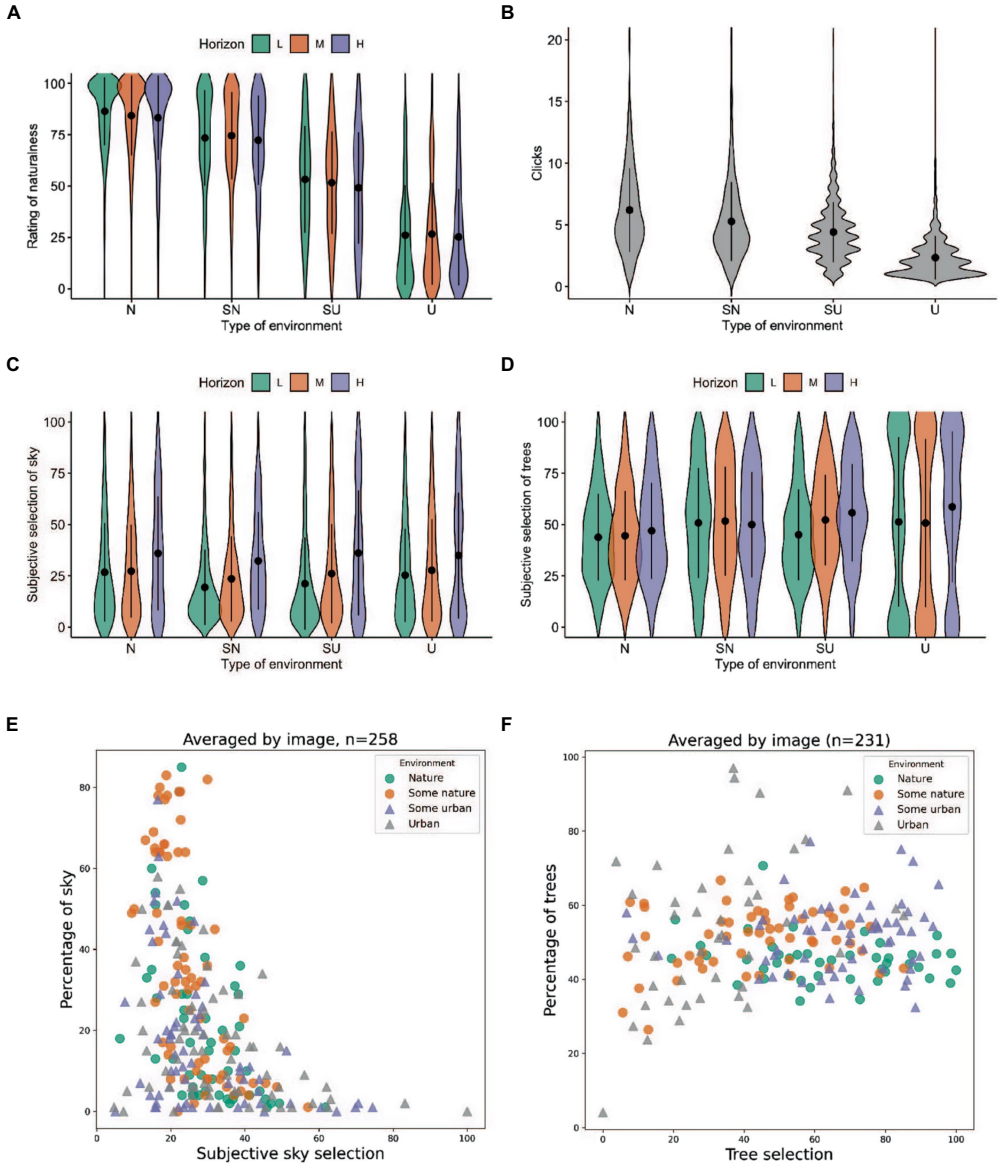
Results. **(A)** Distribution of ratings of naturalness over the experimental conditions type of environment and horizon level. **(B)** Distribution of number of clicks made in each type of environment. **(C)** Distribution of subjective selections of sky over experimental conditions. **(D)** Distribution of subjective selections of trees over experimental conditions. **(E)** Averaged over images, subjective selections of sky plotted against objective visibility of the sky. **(F)** Averaged over images, subjective selections of trees plotted against objective visibility of the sky. **(E,F)** Values are split by the types of environment.

**Figure 3:**
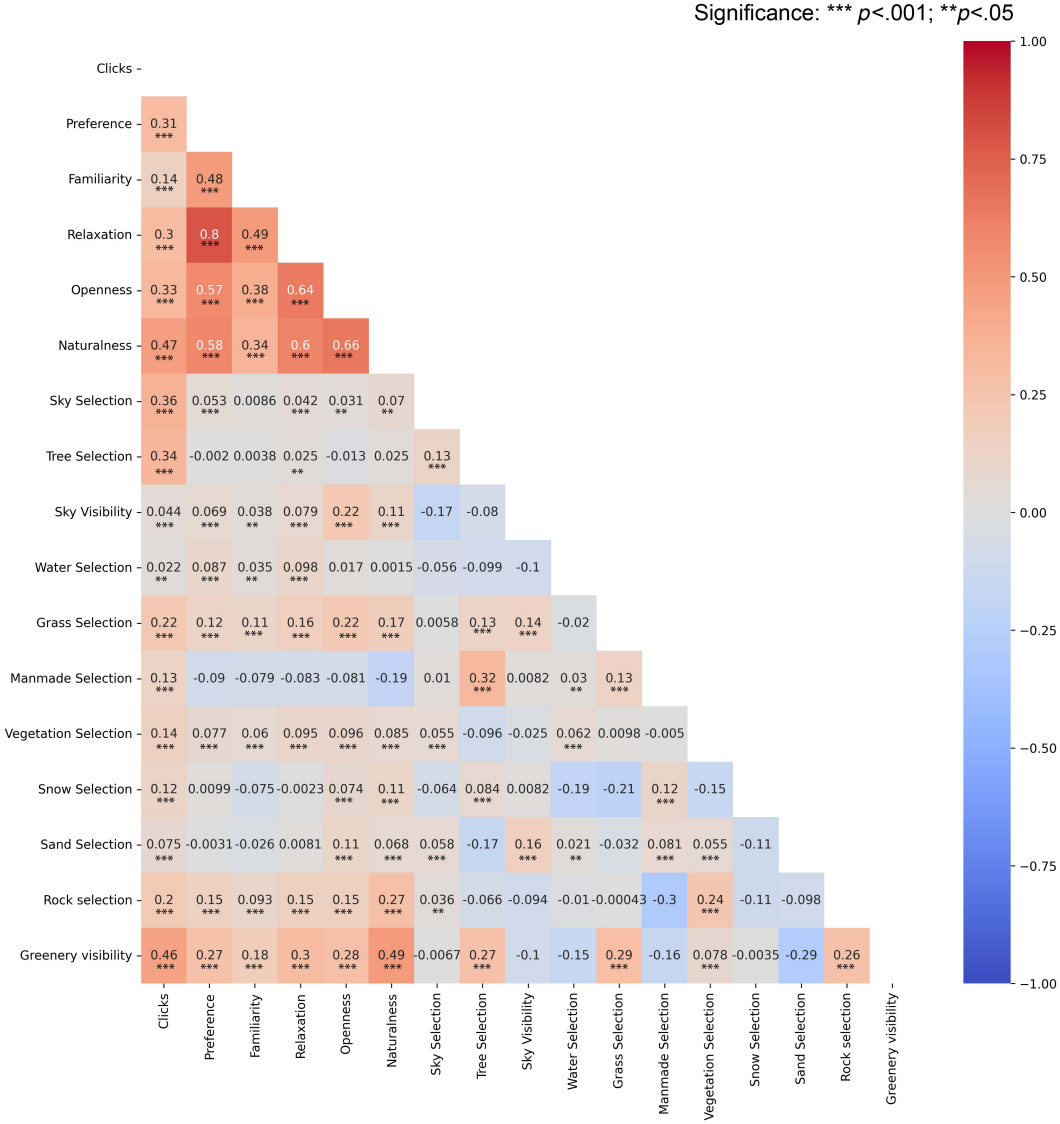
Pairwise correlation heat map.

The non-parametric Kruskal–Wallis test explored the main free-selection task assumption that type of environment has an effect on the number of nature selections. The outcome indicates the statistically significant effect of the type of environment on the average number of nature selections [*H* (3) = 212.18, *p* < 0.001]. Dunn’s *post hoc* multiple comparison test with Bonferroni correction additionally showed the difference is statistically significant (*p* < 0.001) among all environments with Nature (*M* = 6, *SD* = 3) and Some Nature (*M* = 5, *SD* = 3) conditions significantly higher than Urban (*M* = 2, *SD* = 1.5) and Some Urban (*M* = 4, *SD* = 2). We have also scrutinized the effect of the type of environment on ratings of naturalness. Here the Kruskal–Wallis test also showed a significant effect on the rating of naturalness [*H* (3) = 2928.36, *p* < 0.001]. Dunn’s *post hoc* multiple comparison test with Bonferroni correction indicated significant differences in ratings among all conditions with the most substantial difference between Nature (*M* = 84.86, *SD* =18.61) and Urban (*M* = 26.05, *SD* =24.32) (Figure 2B). Moreover, Spearman rho indicated a strong positive correlation (*r* = 0.787, *p* < 0.001, see Figure 3) between the rating of naturalness and the objective amount of nature in the image. Overall, the results show that the main objectives of the free-selection task were met and participants performed the task as expected, making more selections in natural environments than in an urban context.

### Sky in nature-selection context

We investigated how subjective selection of sky was connected to the selection of other semantic characteristics visible on the images. The overview (Table 1, for details see Supplementary material: Selection summary statistics) of the result shows that generally, the highest relative average selection rates were made on Vegetation (*M* = 56, *SD* = 26) and Water (*M* = 53, *SD* = 27). As expected, a lower selection rate was identified in Manmade label (*M* = 29, *SD* = 23), while the lowest selection rate was indicated by Sky (*M* = 27, *SD* = 25). The high standard deviation is understandable and indicated a large dispersal rate between the scores hinted at during the normality diagnostics. Distribution across the experimental conditions shows a more comprehensive picture suggesting greater differences between the horizon level conditions.

**Table 1:** Summary statistics of each label subjective selection (Score EDB).

	Sky	Trees	Water	Grass	Vegetation	Snow	Sand	Rocks	Manmade
Samples	6,397	5,070	950	1,261	616	547	362	357	854
Mean	27	50	53	52	56	39	37	44	29
Std.	25	28	27	38	26	25	23	24	23
Min	0	0	0	6	0	4	3	0	0
25%	7	29	33	29	36	20	19	26	12
50%	19	49	50	45	54	33	32	41	22
75%	41	70	73	69	74	51	52	60	40
Max.	100	100	100	776	100	164	100	100	100

The statistics were calculated based on the samples of pictures where the particular label was present.

### Subjective ratings

We investigated the relationships between the key obtained variables: subjective ratings, objective measure of the sky, number of selections and selection of labels as nature (for summary statistics see Supplementary material: Subjective ratings summary statistics.). Figure 3 shows that the associations indicated significant (*p* < 0.001) moderate to strong positive relationships between all scales. The strongest relationship was detected between the preference rating and emotional restoration with *r* = 0.796 (*p* < 0.001) indicating that the participants were more likely to prefer images that invoked a stronger feeling of relaxation. The increased feeling of relaxation was also strongly correlated with the openness of the space (*r* = 0.638, *p* < 0.001) and higher naturalness ratings (*r* = 0.600, *p* < 0.001). We also found a strong positive association between higher naturalness ratings and preference (*r* = 0.580, *p* < 0.001) and a strong positive relationship between higher naturalness ratings and higher perception of the openness of the space (*r* = 0.600, *p* < 0.001). Rating of familiarity with the scene had a borderline strong positive association with an increased feeling of relaxation (*r* = 0.495, *p* < 0.001), moderate with a rating of openness (*r* = 0.384, *p* < 0.000) and a higher rating of naturalness (*r* = 0.344, *p* < 0.001).

Afterward, we investigated the relationship between the subjective ratings and the objective measure of the presence of sky and greenery. The increased visibility of the sky had a weak positive correlation with ratings of naturalness (*r* = 0.113, *p* < 0.001). On the other hand, the overall visibility of greenery had a borderline strong positive association with increased naturalness ratings (*r* = 0.490, *p* < 0.001). Interestingly, correlations also showed a comparable positive association between the objective amount of greenery and sky on an increased rating of openness of the space (greenery: *r* = 0.278, *p* < 0.001; sky: *r* = 0.220, *p* < 0.001). We also observed that the positive association with an increased feeling of relaxation is stronger for higher visibility of greenery (*r* = 0.303, *p* < 0.001) than the sky (*r* = 0.079, *p* < 0.001). The same pattern was observed in ratings of preference where higher preference ratings were moderately positively correlated with higher visibility of greenery (*r* = 0.270, *p* < 0.001) but we found no association with visibility of the sky (*r* = 0.069, *p* < 0.001).

Furthermore, we found a weak positive association between the subjective rating of naturalness and subjective selection (EDB scores) of key features: sky (*r* = 0.070, *p* < 0.001), trees (*r* = 0.025, *p* < 0.05), and water (*r* = 0.001, *p* = 0.453). Interestingly, at the same time, we found a significant moderate association between a higher number of clicks made on an image and higher sky selection (*r* = 0.361, *p* < 0.001) with a comparable pattern for higher tree selection (*r* = 0.341, *p* < 0.001).

### Effect of environmental context on perception of sky as nature

**Figure 4:**
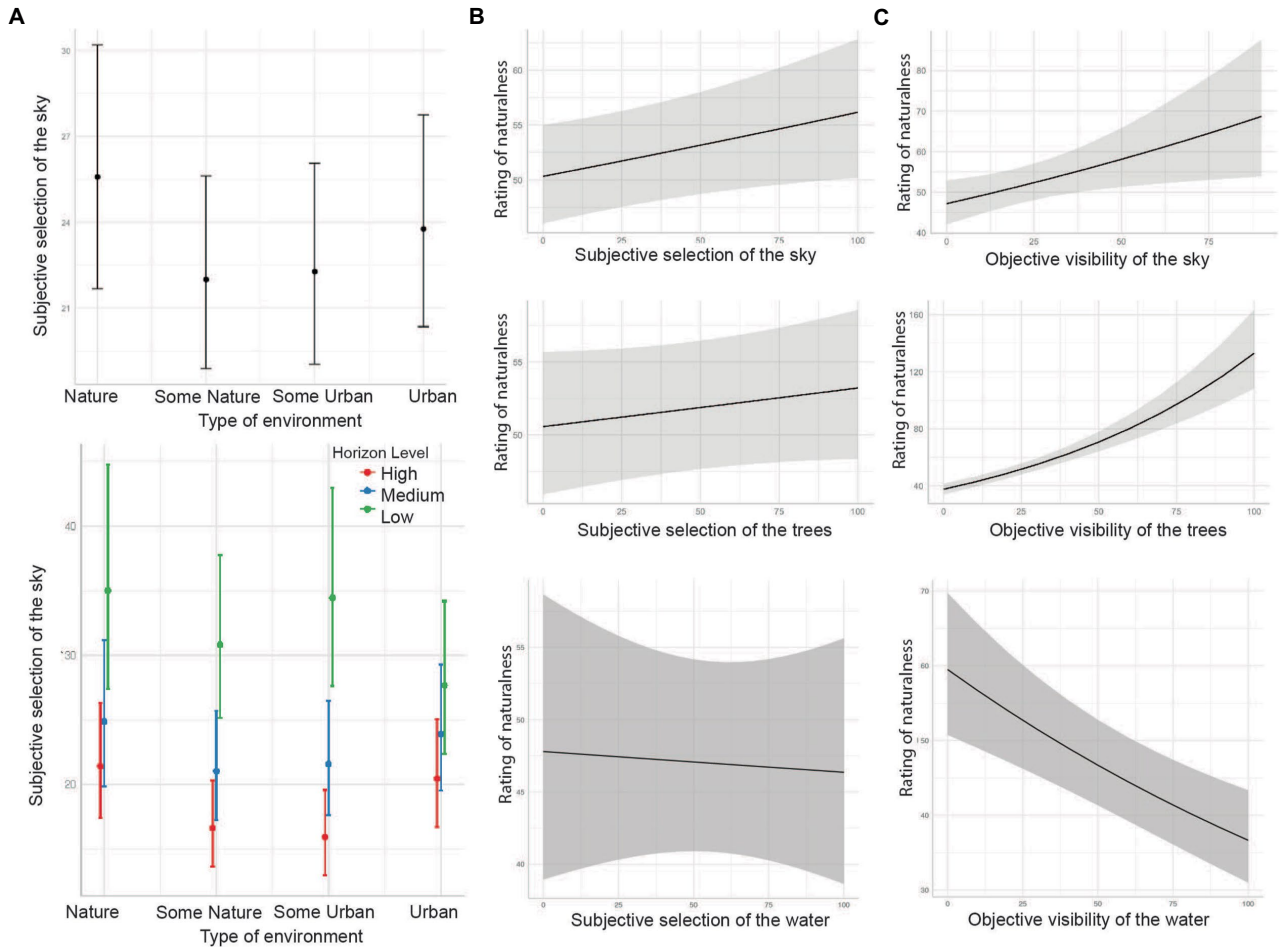
Results of the GLMMs. **(A)** Plot of predicted marginal effect of type of environment on subjective selection of the sky. Below, distribution of the effect by type of environment and horizon. **(B,C)** Modeled predicted marginal effects for subjective and objective selection of sky, trees and water.

To quantify how the selection of sky as nature is associated with the environmental context, we fitted GLMMs for subjective selection of the sky with a stepwise methodology. We assume that the perception of sky as nature depends on its visibility, context and state of the environment. In the initial model, we included the fixed effect of the type of environment as the main (first-level) experimental factor, with the random intercept of participant and image: *EDB_sky_ ~ environment + (1|participant) + (1|image*). The plot of predicted values indicated that sky selections were more likely in natural and urban environments than in mixed (Figure 4A). However, the fitted model estimates indicated that the environment type alone did not significantly affect the selection of sky as nature (*p* > 0.05; see model table in Extended Data). Moreover, marginal *R*^2^ at 0.004 shows that type of environment explains little variance within the model performance (conditional *R^2^* = 0.534). The alternative model extended the additive term for second-level experimental factor of horizon level (*EDB_sky_ ~ environment + horizon + (1|participant) + (1|image)*) to reflect the general experimental design. That factor was associated with the amount of sky present in the image and was presumed to impact the selection. The model’s estimates revealed a statistically insignificant effect of types of environments (*p* > 0.05) but significant (*p* < 0.001) association for each horizon level (for details, see model table in Supplementary material: Extended data. GLMM Tables.). A notable difference was found between the Low horizon, and High horizon condition with sky selection significantly increased when the horizon level was high up in the image, so that the fewer sky was visible the more it was selected as nature (Figure 4A). The marginal *R*^2^ = 0.056 indicated an increase in the explanatory value of the horizon as compared to that of the entire model (conditional *R*^2^_alt_ = 0.533). The difference between baseline and alternative models was significant [*X^2^*(2) = 47, *p* < 0.001]. The improved AIC indicator of the alternative model (AIC_null_ = 53655.2 vs. AIC_alt_ = 53601.6) reveals that the selection of sky as nature depends on more complex associations between variables. Furthermore, the horizon level plays a significant role in rating sky as nature.

Pursuing the relationship between the selection of sky and the horizon level, we reformulated the model regarding sky conditions. As a result, in the second set of models, the model dropped the type of environment as a fixed effect and included horizon level with a random intercept of participant and image name: *EDB_sky_ ~ horizon + (1|participant) + (1|image)*. Model estimates indicate a strong significant effect of all horizon levels (*p* < 0.001). The variance explained within the model was higher than that of the type of environment (marginal *R^2^* = 0.049 and conditional *R^2^* = 0.533)—however, the model’s AIC = 53601.9 was relatively comparable to the previous model. Thus, we added to the model the effect of weather conditions (blue sky, cloudy or grey sky) with an alternative model: *EDB_sky_ ~ horizon + sky conditions + (1|participant) + (1|image).* While the horizon effect remained statistically significant (*p* < 0.001), the grey sky condition was associated with the highest sky selection rate, the blue sky condition the lowest sky selection, and the cloudy sky was marginally significantly different from the grey sky (*p* = 0.046). The sky conditions contributed (marginal *R*^2^_alt_ = 0.064) to the increased explanation of fixed effects of the entire model (conditional *R^2^*_alt_ = 0.532). When compared, the AIC of the alternative model indicated general fit improvement with AIC = 53,588, and the likelihood ratio test suggested better overall performance [*X*^2^(2) = 18.39, *p* < 0.001]. Furthermore, we have investigated the interactive effect between the horizon and sky conditions: EDB_sky_ ~ horizon*sky condition + (1|sub) + (1|image). However, the model estimates indicated no significant interaction between the levels of horizon and conditions in the sky (*p* > 0.05) and the total variance explained by the interaction remained unchanged (marginal *R^2^* = 0.065 and conditional *R^2^* = 0.532). The model’s AIC indicator was higher than that of the previous model (AIC = 53,593), and the likelihood ratio test revealed no significant difference between the models [*X^2^*(2) = 2.51, *p* = 0.643]. Therefore, we assumed that modelling the additive effect of horizon and sky conditions better explained the sky selections.

In the next step, we wanted to account for different conditions determining the state of the environment. We added to the model the variable describing the year’s season: *EDB_sky_ ~ horizon + sky conditions + season + (1|participant) + (1|image).* The estimates predicted that autumn and winter in the image increased the likelihood of selecting sky as nature (see Figure 4A). The spring/summer was the only season statistically insignificant (*p* > 0.05). Moreover, the direct comparison of models indicated that despite an improvement in the AIC parameter (AIC_alt_ = 53,587), the difference between the models was statistically insignificant [*X*^2^(2) = 4.48, *p* = 0.106], and the additive term did not substantially contribute to the explanation of fixed effects variance or overall model (marginal *R^2^* = 0.067 and conditional *R^2^* = 0.532). However, due to the close association between sky conditions and season, we investigated the expected interaction between these two (EDB_sky_ ~ horizon + sky conditions * season + (1|participant) + (1|image)). The estimates showed a significant effect of all interactions. The results indicate that the autumn and grey sky condition predicted the highest rate of sky selection as nature. The grey sky also predicted the highest selections in spring/summer, but the blue sky elicited the highest number of selections in winter. Since the model showed improved performance (AIC_alt_ = 53,566), we tested the differences with the model of the effect of horizon and sky conditions. The interaction also seemed to explain a bigger part of the fixed effects in the model (marginal *R^2^* = 0.087 and conditional *R^2^* = 0.531). The likelihood ratio test showed a significantly better performance of the interaction model [*X^2^*(2) = 34.004, *p* < 0.001]. Therefore, we consider this as the final model.

### Sky affects judgment of naturalness

To test the hypothesis that perceiving sky as nature affects the subjective rating of naturalness, we fitted a GLMM model with naturalness ratings as the outcome. We sought first to explain the association between the subjective sky selection and rating as well as to compare its performance with the model of subjective tree selection. Then, we modelled the association between objective visibility of the sky, trees and the naturalness ratings. The aim of the analysis was to assess the similarity between subjective selection and the general effect of the objective label presence.

The baseline model treated sky selections as a fixed effect. We have used the same parameters for subjective tree selection scores (EDB trees). Our assumption was that the effect of sky selection as nature should be similar to or greater than the subjective selection of trees as nature. For this purpose, we limited the dataset to the images where both trees and sky were present (trees and sky >0, n_images_ = 203, *n* = 5,071). In this manner, we circumvented the problem of zero-inflation in EDB tree scores inside the model, caused by the absence of a particular label on the image. The analysis was performed stepwise, with the same model parameters as the previous models except for adding the Adaptive Gauss-Hermite Quadrature (AGQ) as the maximum log-likelihood term approximation. AGQ is commonly used in approximation of the intractable integrals with normal random effects. It is used as a viable alternative to Laplace approximation method (Jin and Andersson, 2020).

First, we fitted a GLMM for naturalness rating with fixed effect of EDBsky: *Naturalness ~ EDBsky + (1|participant) + (1|image)*. The model’s diagnostics indicated mild underdispersion of residuals, but this did not seem to affect the model significantly. More sky selection as nature was found to be significantly positively associated with the rating of naturalness (*p* < 0.05) (Figure 4B). However, the overall contribution of the score to the explanation of the model’s variance was minimal (marginal *R^2^*_sky_ = 0.001 and conditional *R^2^*_sky_ = 0.422). Next, we performed the same procedure with EDB trees as a fixed effect (*Naturalness ~EDBtrees + (1|participant) + (1|image)*). Interestingly, the effect of trees on the rating of naturalness was found insignificant (*p* = 0.273) with EDB scores not contributing to the explanatory value of the variance in naturalness ratings (marginal *R^2^*_trees_ = 0.000 and conditional *R^2^*_trees_ = 0.423). As a formality, we have compared both models with a likelihood ratio test to confirm that sky scores model performs better than trees scores [*X*^2^(2) = 3.17, *p* < 0.001]. In the last step we investigated if adding the tree selection to the model improved the model’s explanatory value: *Naturalness ~ EDBsky + EDBtrees + (1|participant) +(1|image)*. However, adding tree selection did not significantly improve model performance (marginal *R^2^*_trees_ = 0.001 and conditional *R^2^*_trees_ = 0.422; AIC_alt_ = 49376.6; *X^2^*(1) = 0.49, *p* = 0.484).

To investigate the effect of visibility, we fitted a model for naturalness ratings with experimental variables describing the state of the environment as fixed variables: percentage of sky on the image (instead of horizon, as a direct expression of visibility of the sky), sky conditions and season. The baseline model described the effect of sky visibility on the image on naturalness ratings: *Naturalness ~ percentage of sky + (1|sub) + (1|image).* Generally, the model showed an association between the amount of sky displayed on the image and the rating of naturalness at the significant level (AIC = 49365.9). However, the sky visibility alone only minimally explained the variance within the ratings (marginal *R^2^*_sky_ = 0.011 and conditional *R^2^*_sky_ = 0.422). We have added weather conditions as an explanatory term: *Naturalness ~ percentage of sky + weather conditions + (1|sub) + (1|image)*. However, the effect of weather conditions was found statistically insignificant (*p* > 0.05, marginal *R*^2^_sky_ = 0.013 and conditional *R*^2^_sky_ = 0.422) across the weather conditions and the model overall did not perform significantly better [*X*^2^(2) = 1.06, *p* = 0.588] than baseline. For that reason, we did not retain the weather conditions within the model and proceeded to extend it by the season condition: *Naturalness ~ percentage of sky + season + (1|sub) + (1|image).* Here, the model’s estimates showed a significant effect of winter and spring/summer conditions. Winter and lower sky visibility were found to be associated with increased naturalness ratings. On the other hand, the spring/summer season and low sky visibility were responsible for significantly lower scores. The season was found to moderately contribute to the explanation of fixed effects variance (marginal *R*^2^_sky_ = 0.033 and conditional *R*^2^_sky_ = 0.423) and was responsible for significant improvement in the performance over the baseline model [*X*^2^(2) = 11.77, *p* < 0.05]. In the case of the presence of trees on the image, the GLMM was fit for naturalness ratings with the percentage of trees on the image as a fixed effect: *Naturalness ~ percentage of trees + (1|sub) + (1|image).* As predicted, the baseline model estimates indicated that the visibility of trees on the image played a significant (*p* < 0.001) role in the rating of naturalness. The variance explained by this effect within the model (marginal *R*^2^_trees_ = 0.117 and conditional *R*^2^_trees_ = 0.419) was relatively higher than that of the visibility of the sky and so was the performance of the model [*X*^2^(2) = 70.12, *p* < 0.001]. That alone indicates that the presence of the trees impacted the naturalness ratings more than the presence of the sky. In the Supplementary material: Extended modelling, we have also extended the model for weather and seasonal conditions. Altogether, the model of tree selection indicated that the pattern of effect prediction is better than that of the sky model (Figure 4C).

### The blue and the other blue: Sky and water

To investigate if the two blue spaces: sky and water were associated with the perception of nature to the same degree we have fitted a GLMM model for naturalness ratings with subjective water selection as a fixed variable. For this purpose, we likewise limited the sample size to the images where both water and sky were present (water and sky >0, n_images_ = 73, *n* = 931). Due to significant limitation of the dataset we chose to rerun the subjective sky selection model (*Naturalness ~ EDBsky + (1|participant) + (1|image)*). Consistently, the model predicted significant effect of sky on naturalness (*p* < 0.05), but explained a slightly higher amount of variance (marginal *R^2^*_sky_ = 0.004 and conditional *R^2^*_sky_ = 0.428). The model of subjective water selections (*Naturalness ~ EDBwater + (1|participant) + (1|image)*) indicated that the water selections were not significantly (*p* = 0.823) associated with ratings of naturalness (Figure 4B, marginal *R^2^*_water_ = 0.000 and conditional *R^2^*_water_ = 0.430). Consequently, the direct comparison with likelihood ratio test, confirmed that the subjective sky selections predicted the naturalness ratings better than the selections of water [*X*^2^(0) = 0, *p* < 0.001]. Finally, we have tested if the model with association between naturalness (*Naturalness ~ Water + (1|participant) + (1|image*)) and objective visibility of the water (*p* < 0.001, marginal *R^2^*_water_ = 0.032 and conditional *R^2^*_water_ = 0.372, AIC_water_ = 9,200) was significantly better than that of the objective visibility of the sky (*p* = 0.270, marginal *R^2^*_water_ = 0.001 and conditional *R^2^*_water_ = 0.433, AIC_sky_ = 9,215). The likelihood ratio test showed a significant difference [*X*^2^(0) = 14.86, *p* < 0.001] indicating that the objective water visibility model has better association than that of the objective visibility of the sky.

## Discussion

The main aim of our study was to attempt to close the gap in empirical evidence on the significance of the sky to human perception of nature. We sought to embed the sky as an integral part of the scene, invariant to environment type. In general, participants’ selection of sky was not prioritized when selecting elements of nature, but was represented significantly among selections. Instead, participants focused on elements of greenery present in the landscape, i.e., trees, vegetation. Nevertheless, the sky was clearly selected as nature when available in the image. The subjective selection of sky and the trees were associated to an equal degree with the total number of selections made on the images. The correlations between a subjective rating of naturalness and objective visibility of sky was weaker (but significant) than the correlation with objective visibility of greenery.

More detailed analyses revealed additional interesting findings. The experimental condition of environment type (nature, some nature, some urban, and urban) had no effect on the selection of sky as nature. Although the perception of the sky as nature was independent of the environment in which the choice was made, it depended on the level of horizon (low, medium, and high) on the picture. Interestingly, the less sky was visible in the pictures, the more participants chose sky as an instance of nature. The weather condition on the sky visible on the image seemed to matter, with people being more likely to select the sky as nature when the sky was grey rather than blue or cloudy. This finding is interesting since so far the effect of weather was found to affect the preference for the scene in sunny and bright sky conditions. The effects of these two factors (amount of sky and weather) seem to be independent of each other. However, the weather conditions interacted significantly with the season of the year. We can conclude that in our investigation of whether people consider sky as nature, there is compelling evidence that the perception of sky as nature depended on multiple conditions. The evidence suggests that despite not being strong, the sky was positively associated with the subjective selections of nature and can be considered as an integral part of it. Multiple relationships appeared to explain that the conditions of the environment matter more in considering sky as nature than the type of environment in which sky is presented. The association of subjective sky selection with the visible small amount of the grey sky is particularly intriguing. It is possible that high horizon levels attract participants’ attention for the purpose of orienting participant within the space (Rogers, 1996; Patterson et al., 1997) and therefore unintentionally guiding attention toward the presence of sky (Ooi et al., 2001). Yet, it is difficult to find an explanation for the amplification of the effect in grey sky conditions and the winter season.

Our second hypothesis stated that sky affects the subjective perception of naturalness. In the case of predictability of naturalness ratings by subjective sky selection, the results revealed that sky selection as nature was positively associated with naturalness ratings, unlike the selection of trees and water. This result is in line with earlier evidence, including by Beute and de Kort (2013) who concluded that there is an explicit preference for natural environments with sunny and bright conditions. Moreover, the explanatory value of the sky model did not benefit from the summary effect of subjective selection of the sky and the trees. Interestingly, we found that when we used the objective presence of sky and tree visibility to predict naturalness ratings and objective visibility of the sky remained significant but weak. In contrast, the association between objective visibility of the trees was significantly stronger. Additionally, the association was further modified by weather condition and seasonal changes. Higher naturalness ratings were associated with the objective presence of the sky in winter. Importantly, the presence of the trees affected naturalness ratings more than the presence of the sky. One possible explanation for this could be a subliminal effect of tree presence which is stronger than that of the sky. The presence of trees and vegetation on images has in the previous literature frequently been associated with a high level of non-straight edge density and fractal dimension (Berman et al., 2014; Patuano, 2018; Schertz and Berman, 2019). There is evidence that these spatial properties are associated with the perception of naturalness and that it affects bottom-up visual processing (Kardan et al., 2015).

Lastly, we have compared the naturalness association with a subjective selection of sky and water. The result showed no significant association with subjective water selection but we found subjective sky selection congruent with previous results. However, we observed an association between objective presence of water and ratings of naturalness. To summarize, the subjective sky selection seemed to explain the naturalness ratings better than subjective selection of water, but on the other hand the objective presence of water explained naturalness ratings better than objective presence of the sky. White et al. (2021) explained variances in different associations between blue and greenspaces as a result of overall exposure and accessibility to the space. There is also the possibility that the above-mentioned effect of low-level spatial properties of the water surface may make them less likely to be selected as nature. In the end, we can conclude that the positive association of sky with subjective ratings of naturalness, although weak, extends through subjective and objective measures. Sky results were found to be more consistently linked with naturalness than trees or water. Such results may corroborate the earlier indirect evidence where higher tree cover density, which resulted in more occlusion of the sky, slowed recovery from stress in men (Jiang et al., 2014). Similarly, Gatersleben and Andrews (2013) found that within the natural environment openness of the space, and therefore direct access to sky, was more beneficial to restoration than tree canopy coverage. In their paper, Masoudinejad and Hartig (2018) framed that the restorative quality of the sky can be theoretically framed in ART. They concluded that fascination focused the attentional resources and was influenced by the amount of visible sky. Our evidence suggests that a higher horizon level may guide these resources toward the sky, increasing the frequency with which sky was selected as nature, despite greater visual view to vegetation. Conclusively, we see that sky was considered as nature and played a role in the perception of the environment. However, this role needs further rigorous investigation.

To our knowledge, this is the first study specifically focusing on the place of the sky in the perception and effect of the natural environment. The study benefits from a robust experimental design that aimed to assess the association between sky and the diversity of the environments. We have operationalized the hypotheses by the use of conscious explicit selection of nature as a method of probing the hypothesized connection. There is still a potential in implicit investigation of this relationship by obtaining eye-tracking data to infer attentional processes. Such methods are already in use to investigate the cognitive effects of natural versus urban environments (Stevenson et al., 2019). That way we could directly ascertain if the subjective selection patterns correspond with the implicit effects of the sky.

The study is not without limitations. The experiment was conducted online, during the global pandemic and under lockdown conditions. This might have had some residual effect on the participant’s attitude toward nature. During data analysis, the biggest challenge was a problematic distribution of acquired measurements that required us to employ complex statistical modelling. GLMMs are known for their problematic relationship between model complexity and model performance (Breslow and Clayton, 1993). Moreover, the nature of the dataset also created an issue with overdispersion, underdispersion, and overall problems with the distribution of residuals. It has to be noted that the individual effect of participant and image explains a significant amount of variance across all of the models.

Our main conclusions open new prospects for providing better explanations for the role of environment in mental health and wellbeing. We hope that the encouraging results will motivate further interest in studying the importance of sky in environmental psychology research. Specifically, the possible lines of investigation include the effect of the access to sky, importance in visual selection and a detailed investigation of the sky’s role in urban environments. Ultimately, such knowledge may have the potential to impact urban-design strategies and policymaking.

## Data availability statement

The raw data supporting the conclusions of this article will be made available by the authors, without undue reservation. The materials and methods used to conduct the experiment are available in following repositories: OSF: https://osf.io/w9rcq/ GitHub: https://github.com/PuddleJumper2018/nature_gradient/.

## Ethics statement

The studies involving human participants were reviewed and approved by Local Psychological Ethics Committee at the Center for Psychosocial Medicine (LPEK-0280) at University Medical Center Hamburg-Eppendorf. The patients/participants provided their written informed consent to participate in this study.

## Author contributions

IMS designed and coordinated the study, experimental design, stimuli selection and preprocessing, performed the main data analysis and modelling, prepared figures and wrote the manuscript. AÖ coordinated the study, collected the data, and performed paradigm validation. SS designed and coordinated study, experimental design and stimuli selection, and reviewed the manuscript. SK had the idea for the study, reviewed the design, supervised data acquisition and analysis, and reviewed the manuscript. All authors contributed to the article and approved the submitted version.

## Conflict of interest

The authors declare that the research was conducted in the absence of any commercial or financial relationships that could be construed as a potential conflict of interest.

## Publisher’s note

All claims expressed in this article are solely those of the authors and do not necessarily represent those of their affiliated organizations, or those of the publisher, the editors and the reviewers. Any product that may be evaluated in this article, or claim that may be made by its manufacturer, is not guaranteed or endorsed by the publisher.

## Footnotes

^1^https://polyhaven.com/hdris previously https://hdrihaven.com/hdris accessed 10/12/2020.

^2^ https://helpx.adobe.com/photoshop/using/create-panoramic-images-photomerge.html

^3^ https://www.python.org/
