# Supplementary material for: The other blue: Role of sky in the perception of nature
Source: Front Psychol. 2022 Oct 28;13:932507. doi: 10.3389/fpsyg.2022.932507 (PMC9651055; doi:10.3389/fpsyg.2022.932507)
Supplement: Supplementary file 1 [file Data_Sheet_1.pdf]

**Model: EDBsky ~ factor(Env) + (1|sub) + (1|image)**

| | EDBsky | | | | | | | |
|---|---|---|---|---|---|---|---|---|
| *Predictors* | *Incidence Rate Ratios* | *std. Error* | *std. Beta* | *standardized std. Error* | *CI* | *standardized CI* | *Statistic* | *p* |
| (Intercept) | 23.77 | 1.88 | 23.77 | 1.88 | 20.35 – 27.75 | 20.35 – 27.75 | 40.06 | **<0.001** |
| Env [N] | 1.08 | 0.10 | 1.08 | 0.10 | 0.90 – 1.28 | 0.90 – 1.28 | 0.82 | 0.414 |
| Env [SN] | 0.93 | 0.08 | 0.93 | 0.08 | 0.79 – 1.09 | 0.79 – 1.09 | -0.92 | 0.356 |
| Env [SU] | 0.94 | 0.08 | 0.94 | 0.08 | 0.79 – 1.11 | 0.79 – 1.11 | -0.75 | 0.451 |
| **Random Effects** | | | | | | | | |
| $\sigma^2$ | 0.43 | | | | | | | |
| $\tau_{00\ image}$ | 0.22 | | | | | | | |
| $\tau_{00\ sub}$ | 0.28 | | | | | | | |
| ICC | 0.53 | | | | | | | |
| $N_{sub}$ | 105 | | | | | | | |
| $N_{image}$ | 258 | | | | | | | |
| Observations | 6397 | | | | | | | |
| Marginal $R^2$ / Conditional $R^2$ | 0.004 / 0.534 | | | | | | | |

**Model: EDBsky ~ Env + Horizon + (1|sub) + (1|image)**

| | EDBsky | | | | | | | |
|---|---|---|---|---|---|---|---|---|
| *Predictors* | *Incidence Rate Ratios* | *std. Error* | *std. Beta* | *standardized std. Error* | *CI* | *standardized CI* | *Statistic* | *p* |
| (Intercept) | 23.04 | 1.92 | 23.04 | 1.92 | 19.57 – 27.12 | 19.57 – 27.12 | 37.68 | **<0.001** |
| Env [N] | 1.11 | 0.09 | 1.11 | 0.09 | 0.95 – 1.30 | 0.95 – 1.30 | 1.31 | 0.190 |
| Env [SN] | 0.92 | 0.07 | 0.92 | 0.07 | 0.80 – 1.07 | 0.80 – 1.07 | -1.10 | 0.273 |
| Env [SU] | 0.94 | 0.07 | 0.94 | 0.07 | 0.81 – 1.10 | 0.81 – 1.10 | -0.74 | 0.458 |
| Horizon [L] | 0.81 | 0.05 | 0.81 | 0.05 | 0.71 – 0.92 | 0.71 – 0.92 | -3.26 | **0.001** |
| Horizon [H] | 1.39 | 0.10 | 1.39 | 0.10 | 1.22 – 1.59 | 1.22 – 1.59 | 4.85 | **<0.001** |
| **Random Effects** | | | | | | | | |
| $\sigma^2$ | 0.43 | | | | | | | |
| $\tau_{00\ image}$ | 0.17 | | | | | | | |
| $\tau_{00\ sub}$ | 0.28 | | | | | | | |
| ICC | 0.51 | | | | | | | |
| $N_{sub}$ | 105 | | | | | | | |
| $N_{image}$ | 258 | | | | | | | |
| Observations | 6397 | | | | | | | |
| Marginal $R^2$ / Conditional $R^2$ | 0.056 / 0.533 | | | | | | | |

**Model: EDBsky ~ Horizon + (1|sub) + (1|image)**

| | EDBsky | | | | | | | |
|---|---|---|---|---|---|---|---|---|
| *Predictors* | *Incidence Rate Ratios* | *std. Error* | *std. Beta* | *standardized std. Error* | *CI* | *standardized CI* | *Statistic* | *p* |
| (Intercept) | 22.67 | 1.58 | 22.67 | 1.58 | 19.76 – 25.99 | 19.76 – 25.99 | 44.65 | **<0.001** |
| Horizon [L] | 0.81 | 0.05 | 0.81 | 0.05 | 0.71 – 0.93 | 0.71 – 0.93 | -3.13 | **0.002** |
| Horizon [H] | 1.39 | 0.10 | 1.39 | 0.10 | 1.21 – 1.59 | 1.21 – 1.59 | 4.75 | **<0.001** |
| **Random Effects** | | | | | | | | |
| $\sigma^2$ | 0.43 | | | | | | | |
| $\tau_{00\ image}$ | 0.17 | | | | | | | |
| $\tau_{00\ sub}$ | 0.28 | | | | | | | |
| ICC | 0.51 | | | | | | | |
| $N_{sub}$ | 105 | | | | | | | |
| $N_{image}$ | 258 | | | | | | | |
| Observations | 6397 | | | | | | | |
| Marginal $R^2$ / Conditional $R^2$ | 0.049 / 0.533 | | | | | | | |

**Model: EDBsky ~ Horizon + Sky_condition + (1|sub) + (1|image)**

| | EDBsky | | | | | | | |
|---|---|---|---|---|---|---|---|---|
| *Predictors* | *Incidence Rate Ratios* | *std. Error* | *std. Beta* | *standardized std. Error* | *CI* | *standardized CI* | *Statistic* | *p* |
| (Intercept) | 26.95 | 2.28 | 26.95 | 2.28 | 22.83 – 31.81 | 22.83 – 31.81 | 38.91 | **<0.001** |
| Horizon [L] | 0.81 | 0.05 | 0.81 | 0.05 | 0.71 – 0.92 | 0.71 – 0.92 | -3.34 | **0.001** |
| Horizon [H] | 1.39 | 0.09 | 1.39 | 0.09 | 1.22 – 1.59 | 1.22 – 1.59 | 4.97 | **<0.001** |
| Sky condition [blue] | 0.74 | 0.05 | 0.74 | 0.05 | 0.65 – 0.85 | 0.65 – 0.85 | -4.28 | **<0.001** |
| Sky condition [cloudy] | 0.87 | 0.06 | 0.87 | 0.06 | 0.76 – 1.00 | 0.76 – 1.00 | -1.99 | **0.046** |
| **Random Effects** | | | | | | | | |
| $\sigma^2$ | 0.43 | | | | | | | |
| $\tau_{00\ image}$ | 0.16 | | | | | | | |
| $\tau_{00\ sub}$ | 0.28 | | | | | | | |
| ICC | 0.50 | | | | | | | |
| $N_{sub}$ | 105 | | | | | | | |
| $N_{image}$ | 258 | | | | | | | |
| Observations | 6397 | | | | | | | |
| Marginal $R^2$ / Conditional $R^2$ | 0.064 / 0.532 | | | | | | | |

**Model: EDBsky ~ Horizon*Sky_condition + (1|sub) + (1|image)**

| | EDBsky | | | | | | | |
|---|---|---|---|---|---|---|---|---|
| *Predictors* | *Incidence Rate Ratios* | *std. Error* | *std. Beta* | *standardized std. Error* | *CI* | *standardized CI* | *Statistic* | *p* |
| (Intercept) | 27.46 | 2.98 | 27.46 | 2.98 | 22.20 – 33.98 | 22.20 – 33.98 | 30.50 | **<0.001** |
| Horizon [L] | 0.78 | 0.10 | 0.78 | 0.10 | 0.61 – 1.01 | 0.61 – 1.01 | -1.89 | 0.059 |
| Horizon [H] | 1.36 | 0.20 | 1.36 | 0.20 | 1.03 – 1.81 | 1.03 – 1.81 | 2.15 | **0.031** |
| Sky condition [blue] | 0.74 | 0.09 | 0.74 | 0.09 | 0.59 – 0.94 | 0.59 – 0.94 | -2.49 | **0.013** |
| Sky condition [cloudy] | 0.82 | 0.10 | 0.82 | 0.10 | 0.65 – 1.04 | 0.65 – 1.04 | -1.61 | 0.107 |
| Horizon [L] * Sky condition [blue] | 1.05 | 0.17 | 1.05 | 0.17 | 0.76 – 1.45 | 0.76 – 1.45 | 0.30 | 0.763 |
| Horizon [H] * Sky condition [blue] | 0.93 | 0.17 | 0.93 | 0.17 | 0.66 – 1.32 | 0.66 – 1.32 | -0.40 | 0.692 |
| Horizon [L] * Sky condition [cloudy] | 1.04 | 0.17 | 1.04 | 0.17 | 0.75 – 1.44 | 0.75 – 1.44 | 0.23 | 0.820 |
| Horizon [H] * Sky condition [cloudy] | 1.14 | 0.20 | 1.14 | 0.20 | 0.80 – 1.61 | 0.80 – 1.61 | 0.71 | 0.480 |
| **Random Effects** | | | | | | | | |
| $\sigma^2$ | 0.43 | | | | | | | |
| $\tau_{00\ image}$ | 0.16 | | | | | | | |
| $\tau_{00\ sub}$ | 0.28 | | | | | | | |
| ICC | 0.50 | | | | | | | |
| $N_{sub}$ | 105 | | | | | | | |
| $N_{image}$ | 258 | | | | | | | |
| Observations | 6397 | | | | | | | |
| Marginal $R^2$ / Conditional $R^2$ | 0.065 / 0.532 | | | | | | | |

**Model: EDBsky ~ Horizon * Sky_condition + Season + (1|sub) + (1|image)**

| | EDBsky | | | | | | | |
|---|---|---|---|---|---|---|---|---|
| *Predictors* | *Incidence Rate Ratios* | *std. Error* | *std. Beta* | *standardized std. Error* | *CI* | *standardized CI* | *Statistic* | *p* |
| (Intercept) | 27.81 | 2.46 | 27.81 | 2.46 | 23.38 – 33.06 | 23.38 – 33.06 | 37.63 | **<0.001** |
| Horizon [L] | 0.81 | 0.05 | 0.81 | 0.05 | 0.71 – 0.92 | 0.71 – 0.92 | -3.36 | **0.001** |
| Horizon [H] | 1.39 | 0.09 | 1.39 | 0.09 | 1.22 – 1.58 | 1.22 – 1.58 | 4.98 | **<0.001** |
| Sky condition [blue] | 0.75 | 0.05 | 0.75 | 0.05 | 0.65 – 0.87 | 0.65 – 0.87 | -3.98 | **<0.001** |
| Sky condition [cloudy] | 0.86 | 0.06 | 0.86 | 0.06 | 0.75 – 0.99 | 0.75 – 0.99 | -2.08 | **0.038** |
| Season [Spring/Summer] | 0.97 | 0.06 | 0.97 | 0.06 | 0.87 – 1.09 | 0.87 – 1.09 | -0.49 | 0.622 |
| Season [Winter] | 0.82 | 0.08 | 0.82 | 0.08 | 0.68 – 0.99 | 0.68 – 0.99 | -2.11 | **0.035** |
| **Random Effects** | | | | | | | | |
| $\sigma^2$ | 0.43 | | | | | | | |
| $\tau_{00\ image}$ | 0.15 | | | | | | | |
| $\tau_{00\ sub}$ | 0.28 | | | | | | | |
| ICC | 0.50 | | | | | | | |
| $N_{sub}$ | 105 | | | | | | | |
| $N_{image}$ | 258 | | | | | | | |
| Observations | 6397 | | | | | | | |
| Marginal $R^2$ / Conditional $R^2$ | 0.067 / 0.532 | | | | | | | |

**Model: EDBsky ~ Horizon + Sky_condition * Season + (1|sub) + (1|image)**

| | EDBsky | | | | | | | |
|---|---|---|---|---|---|---|---|---|
| *Predictors* | *Incidence Rate Ratios* | *std. Error* | *std. Beta* | *standardized std. Error* | *CI* | *standardized CI* | *Statistic* | *p* |
| (Intercept) | 33.15 | 3.15 | 33.15 | 3.15 | 27.51 – 39.95 | 27.51 – 39.95 | 36.81 | **<0.001** |
| Horizon [L] | 0.80 | 0.05 | 0.80 | 0.05 | 0.71 – 0.90 | 0.71 – 0.90 | -3.73 | **<0.001** |
| Horizon [H] | 1.40 | 0.09 | 1.40 | 0.09 | 1.24 – 1.59 | 1.24 – 1.59 | 5.44 | **<0.001** |
| Sky condition [blue] | 0.49 | 0.05 | 0.49 | 0.05 | 0.40 – 0.61 | 0.40 – 0.61 | -6.58 | **<0.001** |
| Sky condition [cloudy] | 0.73 | 0.07 | 0.73 | 0.07 | 0.61 – 0.88 | 0.61 – 0.88 | -3.34 | **0.001** |
| Season [Spring/Summer] | 0.72 | 0.08 | 0.72 | 0.08 | 0.58 – 0.90 | 0.58 – 0.90 | -2.90 | **0.004** |
| Season [Winter] | 0.43 | 0.08 | 0.43 | 0.08 | 0.31 – 0.61 | 0.31 – 0.61 | -4.79 | **<0.001** |
| Sky condition [blue] * Season [Spring/Summer] | 1.81 | 0.27 | 1.81 | 0.27 | 1.36 – 2.42 | 1.36 – 2.42 | 4.03 | **<0.001** |
| Sky condition [cloudy] * Season [Spring/Summer] | 1.32 | 0.19 | 1.32 | 0.19 | 1.01 – 1.74 | 1.01 – 1.74 | 2.00 | **0.046** |
| Sky condition [blue] * Season [Winter] | 3.05 | 0.67 | 3.05 | 0.67 | 1.98 – 4.69 | 1.98 – 4.69 | 5.07 | **<0.001** |
| Sky condition [cloudy] * Season [Winter] | 1.79 | 0.44 | 1.79 | 0.44 | 1.11 – 2.89 | 1.11 – 2.89 | 2.37 | **0.018** |
| **Random Effects** | | | | | | | | |
| $\sigma^2$ | 0.43 | | | | | | | |
| $\tau_{00\ image}$ | 0.13 | | | | | | | |
| $\tau_{00\ sub}$ | 0.28 | | | | | | | |
| ICC | 0.49 | | | | | | | |
| $N_{sub}$ | 105 | | | | | | | |
| $N_{image}$ | 258 | | | | | | | |
| Observations | 6397 | | | | | | | |
| Marginal $R^2$ / Conditional $R^2$ | 0.087 / 0.531 | | | | | | | |

**Scale_natural ~ EDBsky + (1|sub) + (1|image)**

| | Scale_natural | | | | | | | |
|---|---|---|---|---|---|---|---|---|
| *Predictors* | *Incidence Rate Ratios* | *std. Error* | *std. Beta* | *standardized std. Error* | *CI* | *standardized CI* | *Statistic* | *p* |
| (Intercept) | 50.33 | 2.28 | 51.82 | 2.22 | 46.05 – 55.02 | 47.64 – 56.37 | 86.33 | **<0.001** |
| EDBsky | 1.00 | 0.00 | 1.03 | 0.01 | 1.00 – 1.00 | 1.00 – 1.05 | 2.08 | **0.037** |
| **Random Effects** | | | | | | | | |
| $\sigma^2$ | 0.42 | | | | | | | |
| $\tau_{00\ image}$ | 0.26 | | | | | | | |
| $\tau_{00\ sub}$ | 0.05 | | | | | | | |
| ICC | 0.42 | | | | | | | |
| $N_{sub}$ | 105 | | | | | | | |
| $N_{image}$ | 203 | | | | | | | |
| Observations | 5070 | | | | | | | |
| Marginal $R^2$ / Conditional $R^2$ | 0.001 / 0.422 | | | | | | | |

**Model: Scale_natural ~ EDBtrees + (1|sub) + (1|image)**

| | Scale_natural | | | | | | | |
|---|---|---|---|---|---|---|---|---|
| *Predictors* | *Incidence Rate Ratios* | *std. Error* | *std. Beta* | *standardized std. Error* | *CI* | *standardized CI* | *Statistic* | *p* |
| (Intercept) | 50.58 | 2.48 | 51.87 | 2.24 | 45.93 – 55.69 | 47.67 – 56.44 | 79.88 | **<0.001** |
| EDBtrees | 1.00 | 0.00 | 1.01 | 0.01 | 1.00 – 1.00 | 0.99 – 1.04 | 1.10 | 0.273 |
| **Random Effects** | | | | | | | | |
| $\sigma^2$ | 0.42 | | | | | | | |
| $\tau_{00\ image}$ | 0.26 | | | | | | | |
| $\tau_{00\ sub}$ | 0.05 | | | | | | | |
| ICC | 0.42 | | | | | | | |
| $N_{sub}$ | 105 | | | | | | | |
| $N_{image}$ | 203 | | | | | | | |
| Observations | 5070 | | | | | | | |
| Marginal $R^2$ / Conditional $R^2$ | 0.000 / 0.423 | | | | | | | |

**Model: Scale_natural ~ EDBsky + EDBtrees + (1|sub) + (1|image)**

| | Scale_natural | | | | | | | |
|---|---|---|---|---|---|---|---|---|
| *Predictors* | *Incidence Rate Ratios* | *std. Error* | *std. Beta* | *standardized std. Error* | *CI* | *standardized CI* | *Statistic* | *p* |
| (Intercept) | 49.57 | 2.48 | 51.80 | 2.23 | 44.94 – 54.69 | 47.61 – 56.36 | 77.96 | **<0.001** |
| EDBsky | 1.00 | 0.00 | 1.03 | 0.01 | 1.00 – 1.00 | 1.00 – 1.05 | 1.91 | 0.056 |
| EDBtrees | 1.00 | 0.00 | 1.01 | 0.01 | 1.00 – 1.00 | 0.98 – 1.04 | 0.71 | 0.478 |
| **Random Effects** | | | | | | | | |
| $\sigma^2$ | 0.42 | | | | | | | |
| $\tau_{00\ image}$ | 0.26 | | | | | | | |
| $\tau_{00\ sub}$ | 0.05 | | | | | | | |
| ICC | 0.42 | | | | | | | |
| $N_{sub}$ | 105 | | | | | | | |
| $N_{image}$ | 203 | | | | | | | |
| Observations | 5070 | | | | | | | |
| Marginal $R^2$ / Conditional $R^2$ | 0.001 / 0.423 | | | | | | | |

**Model: Scale_natural ~ PercentofSky + (1|sub) + (1|image)**

| | Scale_natural | | | | | | | |
|---|---|---|---|---|---|---|---|---|
| *Predictors* | *Incidence Rate Ratios* | *std. Error* | *std. Beta* | *standardized std. Error* | *CI* | *standardized CI* | *Statistic* | *p* |
| (Intercept) | 47.18 | 2.74 | 52.26 | 2.23 | 42.10 – 52.88 | 48.07 – 56.82 | 66.26 | **<0.001** |
| PercentofSky | 1.00 | 0.00 | 1.09 | 0.04 | 1.00 – 1.01 | 1.02 – 1.18 | 2.41 | **0.016** |
| **Random Effects** | | | | | | | | |
| $\sigma^2$ | 0.42 | | | | | | | |
| $\tau_{00\ image}$ | 0.25 | | | | | | | |
| $\tau_{00\ sub}$ | 0.05 | | | | | | | |
| ICC | 0.42 | | | | | | | |
| $N_{sub}$ | 105 | | | | | | | |
| $N_{image}$ | 203 | | | | | | | |
| Observations | 5070 | | | | | | | |
| Marginal $R^2$ / Conditional $R^2$ | 0.011 / 0.422 | | | | | | | |

**Model: Scale_natural ~ PercentofSky + Sky_condition + (1|sub) + (1|image)**

| | Scale_natural | | | | | | | |
|---|---|---|---|---|---|---|---|---|
| *Predictors* | *Incidence Rate Ratios* | *std. Error* | *std. Beta* | *standardized std. Error* | *CI* | *standardized CI* | *Statistic* | *p* |
| (Intercept) | 44.62 | 3.66 | 49.22 | 3.83 | 37.99 – 52.40 | 42.26 – 57.32 | 46.31 | **<0.001** |
| PercentofSky | 1.00 | 0.00 | 1.09 | 0.04 | 1.00 – 1.01 | 1.01 – 1.17 | 2.26 | **0.024** |
| Sky condition [blue] | 1.10 | 0.10 | 1.10 | 0.10 | 0.92 – 1.32 | 0.92 – 1.32 | 1.03 | 0.303 |
| Sky condition [cloudy] | 1.06 | 0.11 | 1.06 | 0.11 | 0.87 – 1.29 | 0.87 – 1.29 | 0.60 | 0.551 |
| **Random Effects** | | | | | | | | |
| $\sigma^2$ | 0.42 | | | | | | | |
| $\tau_{00\ image}$ | 0.25 | | | | | | | |
| $\tau_{00\ sub}$ | 0.05 | | | | | | | |
| ICC | 0.41 | | | | | | | |
| $N_{sub}$ | 105 | | | | | | | |
| $N_{image}$ | 203 | | | | | | | |
| Observations | 5070 | | | | | | | |
| Marginal $R^2$ / Conditional $R^2$ | 0.013 / 0.422 | | | | | | | |

**Model: Scale_natural ~ PercentofSky + Season + (1|sub) + (1|image)**

| | Scale_natural | | | | | | | |
|---|---|---|---|---|---|---|---|---|
| *Predictors* | *Incidence Rate Ratios* | *std. Error* | *std. Beta* | *standardized std. Error* | *CI* | *standardized CI* | *Statistic* | *p* |
| (Intercept) | 59.93 | 6.63 | 66.44 | 6.70 | 48.25 – 74.44 | 54.53 – 80.96 | 37.01 | **<0.001** |
| PercentofSky | 1.00 | 0.00 | 1.09 | 0.04 | 1.00 – 1.01 | 1.02 – 1.17 | 2.48 | **0.013** |
| Season [Spring/Summer] | 0.70 | 0.08 | 0.70 | 0.08 | 0.57 – 0.87 | 0.57 – 0.87 | -3.18 | **0.001** |
| Season [Autumn] | 0.84 | 0.10 | 0.84 | 0.10 | 0.67 – 1.05 | 0.67 – 1.05 | -1.51 | 0.130 |
| **Random Effects** | | | | | | | | |
| $\sigma^2$ | 0.42 | | | | | | | |
| $\tau_{00\ image}$ | 0.23 | | | | | | | |
| $\tau_{00\ sub}$ | 0.05 | | | | | | | |
| ICC | 0.40 | | | | | | | |
| $N_{sub}$ | 105 | | | | | | | |
| $N_{image}$ | 203 | | | | | | | |
| Observations | 5070 | | | | | | | |
| Marginal $R^2$ / Conditional $R^2$ | 0.033 / 0.423 | | | | | | | |

**Model: Scale_natural ~ %trees + (1|sub) + (1|image)**

| | Scale_natural | | | | | | | |
|---|---|---|---|---|---|---|---|---|
| *Predictors* | *Incidence Rate Ratios* | *std. Error* | *std. Beta* | *standardized std. Error* | *CI* | *standardized CI* | *Statistic* | *p* |
| (Intercept) | 37.69 | 1.91 | 52.53 | 1.98 | 34.13 – 41.62 | 48.80 – 56.55 | 71.70 | **<0.001** |
| TreesPercent | 1.01 | 0.00 | 1.34 | 0.04 | 1.01 – 1.02 | 1.26 – 1.42 | 9.56 | **<0.001** |
| **Random Effects** | | | | | | | | |
| $\sigma^2$ | 0.42 | | | | | | | |
| $\tau_{00\ image}$ | 0.17 | | | | | | | |
| $\tau_{00\ sub}$ | 0.05 | | | | | | | |
| ICC | 0.34 | | | | | | | |
| $N_{sub}$ | 105 | | | | | | | |
| $N_{image}$ | 203 | | | | | | | |
| Observations | 5070 | | | | | | | |
| Marginal $R^2$ / Conditional $R^2$ | 0.117 / 0.419 | | | | | | | |

**Model: Scale_natural ~ %trees + Sky_condition + (1|sub) + (1|image)**

| | Scale_natural | | | | | | | |
|---|---|---|---|---|---|---|---|---|
| *Predictors* | *Incidence Rate Ratios* | *std. Error* | *std. Beta* | *standardized std. Error* | *CI* | *standardized CI* | *Statistic* | *p* |
| (Intercept) | 31.84 | 2.42 | 45.09 | 2.91 | 27.44 – 36.96 | 39.73 – 51.18 | 45.55 | **<0.001** |
| TreesPercent | 1.01 | 0.00 | 1.35 | 0.04 | 1.01 – 1.02 | 1.28 – 1.44 | 10.09 | **<0.001** |
| Sky condition [blue] | 1.19 | 0.09 | 1.19 | 0.09 | 1.03 – 1.39 | 1.03 – 1.39 | 2.30 | **0.021** |
| Sky condition [cloudy] | 1.27 | 0.10 | 1.27 | 0.10 | 1.08 – 1.49 | 1.08 – 1.49 | 2.95 | **0.003** |
| **Random Effects** | | | | | | | | |
| $\sigma^2$ | 0.42 | | | | | | | |
| $\tau_{00\ image}$ | 0.16 | | | | | | | |
| $\tau_{00\ sub}$ | 0.05 | | | | | | | |
| ICC | 0.33 | | | | | | | |
| $N_{sub}$ | 105 | | | | | | | |
| $N_{image}$ | 203 | | | | | | | |
| Observations | 5070 | | | | | | | |
| Marginal $R^2$ / Conditional $R^2$ | 0.127 / 0.418 | | | | | | | |

**Model: Scale_natural ~ %trees + Season + sky_condition + (1|sub) + (1|image)**

| | Scale_natural | | | | | | | |
|---|---|---|---|---|---|---|---|---|
| *Predictors* | *Incidence Rate Ratios* | *std. Error* | *std. Beta* | *standardized std. Error* | *CI* | *standardized CI* | *Statistic* | *p* |
| (Intercept) | 40.78 | 4.35 | 57.32 | 5.70 | 33.09 – 50.25 | 47.17 – 69.65 | 34.79 | **<0.001** |
| TreesPercent | 1.01 | 0.00 | 1.35 | 0.04 | 1.01 – 1.02 | 1.27 – 1.43 | 9.98 | **<0.001** |
| Season [Spring/Summer] | 0.73 | 0.07 | 0.73 | 0.07 | 0.61 – 0.88 | 0.61 – 0.88 | -3.40 | **0.001** |
| Season [Autumn] | 0.78 | 0.07 | 0.78 | 0.07 | 0.65 – 0.94 | 0.65 – 0.94 | -2.58 | **0.010** |
| Sky condition [blue] | 1.19 | 0.09 | 1.19 | 0.09 | 1.03 – 1.39 | 1.03 – 1.39 | 2.34 | **0.020** |
| Sky condition [cloudy] | 1.29 | 0.10 | 1.29 | 0.10 | 1.10 – 1.50 | 1.10 – 1.50 | 3.19 | **0.001** |
| **Random Effects** | | | | | | | | |
| $\sigma^2$ | 0.42 | | | | | | | |
| $\tau_{00\ image}$ | 0.15 | | | | | | | |
| $\tau_{00\ sub}$ | 0.05 | | | | | | | |
| ICC | 0.32 | | | | | | | |
| $N_{sub}$ | 105 | | | | | | | |
| $N_{image}$ | 203 | | | | | | | |
| Observations | 5070 | | | | | | | |
| Marginal $R^2$ / Conditional $R^2$ | 0.141 / 0.418 | | | | | | | |

**Model: Scale_natural ~ Sky + Trees + (1|sub) + (1|image)**

| | Scale_natural | | | | | | | |
|---|---|---|---|---|---|---|---|---|
| *Predictors* | *Incidence Rate Ratios* | *std. Error* | *std. Beta* | *standardized std. Error* | *CI* | *standardized CI* | *Statistic* | *p* |
| (Intercept) | 57.77 | 3.06 | 51.84 | 2.24 | 52.08 – 64.08 | 47.64 – 56.41 | 76.66 | **<0.001** |
| Sky | 1.00 | 0.00 | 0.96 | 0.01 | 1.00 – 1.00 | 0.93 – 0.99 | -2.43 | **0.015** |
| Trees | 1.00 | 0.00 | 0.94 | 0.02 | 1.00 – 1.00 | 0.91 – 0.98 | -3.40 | **0.001** |
| **Random Effects** | | | | | | | | |
| $\sigma^2$ | 0.42 | | | | | | | |
| $\tau_{00\ image}$ | 0.26 | | | | | | | |
| $\tau_{00\ sub}$ | 0.05 | | | | | | | |
| ICC | 0.42 | | | | | | | |
| $N_{sub}$ | 105 | | | | | | | |
| $N_{image}$ | 203 | | | | | | | |
| Observations | 5070 | | | | | | | |
| Marginal $R^2$ / Conditional $R^2$ | 0.004 / 0.425 | | | | | | | |

**Scale_natural ~ EDBwater + (1|sub) + (1|image)**

| | Scale_natural | | | | | | | |
|---|---|---|---|---|---|---|---|---|
| *Predictors* | *Incidence Rate Ratios* | *std. Error* | *std. Beta* | *standardized std. Error* | *CI* | *standardized CI* | *Statistic* | *p* |
| (Intercept) | 47.80 | 4.99 | 47.04 | 3.35 | 38.95 – 58.65 | 40.91 – 54.09 | 37.04 | **<0.001** |
| EDBwater | 1.00 | 0.00 | 0.99 | 0.04 | 1.00 – 1.00 | 0.92 – 1.06 | -0.22 | 0.823 |
| **Random Effects** | | | | | | | | |
| $\sigma^2$ | 0.42 | | | | | | | |
| $\tau_{00\ sub}$ | 0.03 | | | | | | | |
| $\tau_{00\ image}$ | 0.29 | | | | | | | |
| ICC | 0.43 | | | | | | | |
| $N_{sub}$ | 103 | | | | | | | |
| $N_{image}$ | 78 | | | | | | | |
| Observations | 950 | | | | | | | |
| Marginal $R^2$ / Conditional $R^2$ | 0.000 / 0.430 | | | | | | | |

**Naturalness ~ Sky selection + (1|participant) + (1|image)_2**

| | Scale_natural | | | | | | | |
|---|---|---|---|---|---|---|---|---|
| *Predictors* | *Incidence Rate Ratios* | *std. Error* | *std. Beta* | *standardized std. Error* | *CI* | *standardized CI* | *Statistic* | *p* |
| (Intercept) | 44.32 | 3.38 | 46.93 | 3.31 | 38.17 – 51.46 | 40.87 – 53.88 | 49.77 | **<0.001** |
| EDBsky | 1.00 | 0.00 | 1.06 | 0.03 | 1.00 – 1.00 | 1.00 – 1.12 | 2.01 | **0.044** |
| **Random Effects** | | | | | | | | |
| $\sigma^2$ | 0.42 | | | | | | | |
| $\tau_{00\ sub}$ | 0.03 | | | | | | | |
| $\tau_{00\ image}$ | 0.28 | | | | | | | |
| ICC | 0.43 | | | | | | | |
| $N_{sub}$ | 103 | | | | | | | |
| $N_{image}$ | 78 | | | | | | | |
| Observations | 950 | | | | | | | |
| Marginal $R^2$ / Conditional $R^2$ | 0.004 / 0.428 | | | | | | | |

**Scale_natural ~ Water + (1|sub) + (1|image)**

| | Scale_natural | | | | | | | |
|---|---|---|---|---|---|---|---|---|
| *Predictors* | *Incidence Rate Ratios* | *std. Error* | *std. Beta* | *standardized std. Error* | *CI* | *standardized CI* | *Statistic* | *p* |
| (Intercept) | 59.49 | 4.81 | 47.18 | 2.92 | 50.78 – 69.70 | 41.79 – 53.28 | 50.57 | **<0.001** |
| Water | 1.00 | 0.00 | 0.86 | 0.03 | 0.99 – 1.00 | 0.81 – 0.92 | -4.38 | **<0.001** |
| **Random Effects** | | | | | | | | |
| $\sigma^2$ | 0.42 | | | | | | | |
| $\tau_{00\ sub}$ | 0.02 | | | | | | | |
| $\tau_{00\ image}$ | 0.20 | | | | | | | |
| ICC | 0.35 | | | | | | | |
| $N_{sub}$ | 103 | | | | | | | |
| $N_{image}$ | 78 | | | | | | | |
| Observations | 950 | | | | | | | |
| Marginal $R^2$ / Conditional $R^2$ | 0.032 / 0.372 | | | | | | | |

**Scale_natural ~ Sky + (1|sub) + (1|image) (concurrent water)**

| | **Scale_natural** | | | | | | | |
|---|---|---|---|---|---|---|---|---|
| *Predictors* | *Incidence Rate Ratios* | *std. Error* | *std. Beta* | *standardized std. Error* | *CI* | *standardized CI* | *Statistic* | *p* |
| (Intercept) | 46.14 | 3.38 | 47.02 | 3.35 | 39.96 – 53.26 | 40.88 – 54.07 | 52.29 | **<0.001** |
| Sky | 1.00 | 0.00 | 1.03 | 0.03 | 1.00 – 1.00 | 0.98 – 1.09 | 1.10 | 0.270 |
| **Random Effects** | | | | | | | | |
| $\sigma^2$ | 0.42 | | | | | | | |
| $\tau_{00\ sub}$ | 0.03 | | | | | | | |
| $\tau_{00\ image}$ | 0.29 | | | | | | | |
| ICC | 0.43 | | | | | | | |
| $N_{sub}$ | 103 | | | | | | | |
| $N_{image}$ | 78 | | | | | | | |
| Observations | 950 | | | | | | | |
| Marginal $R^2$ / Conditional $R^2$ | 0.001 / 0.433 | | | | | | | |
