# Supplementary figures and images for: The other blue: Role of sky in the perception of nature
Source: Front Psychol. 2022 Oct 28;13:932507. doi: 10.3389/fpsyg.2022.932507 (PMC9651055; doi:10.3389/fpsyg.2022.932507)

**Supplement:** Extended data.Distribution plots of key dependents variables:


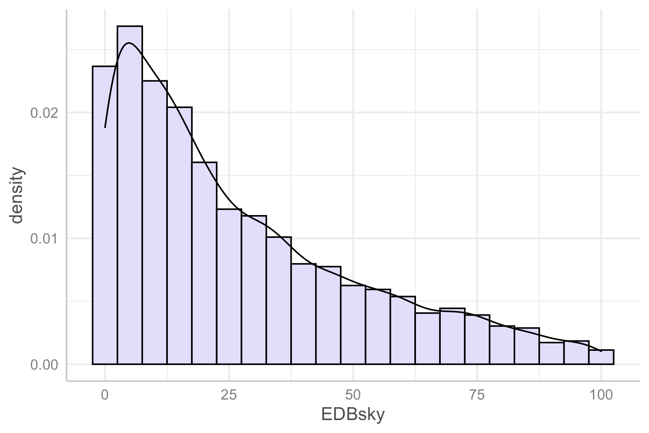

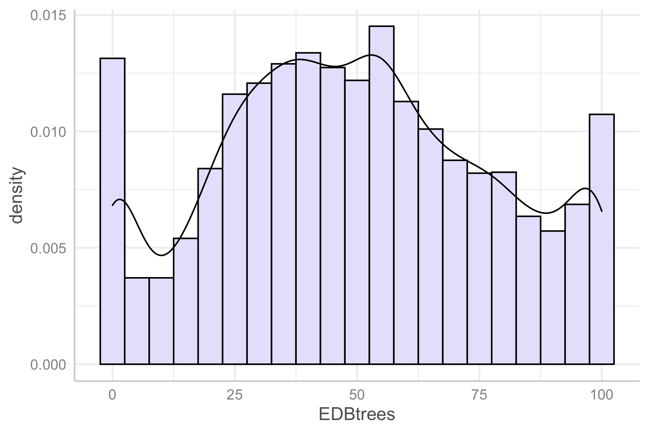

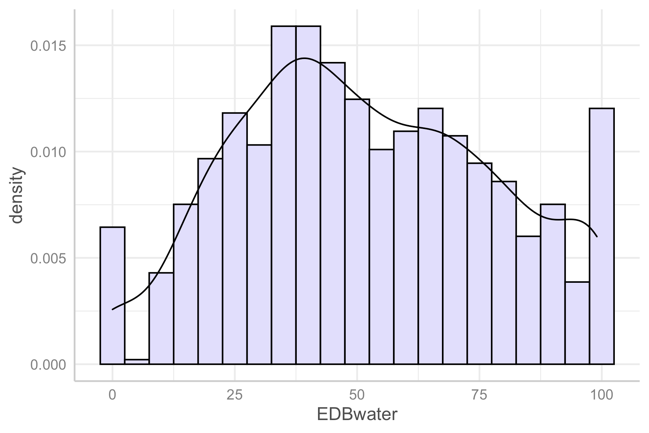

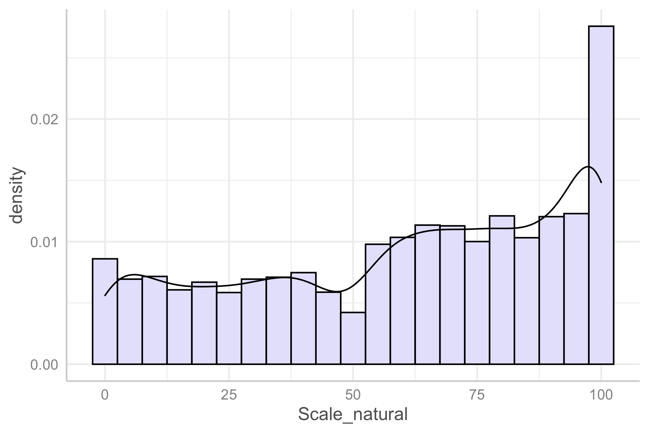

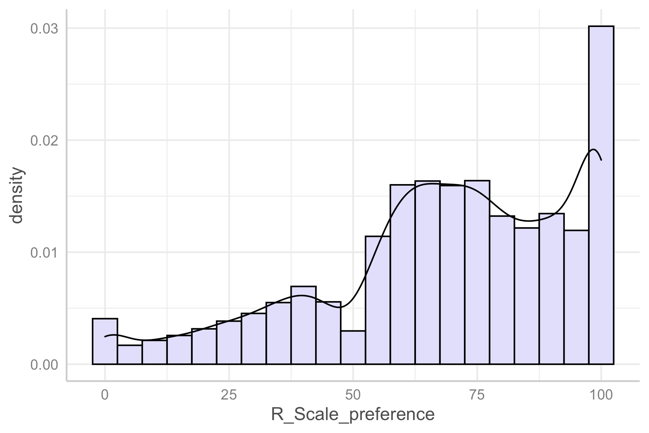

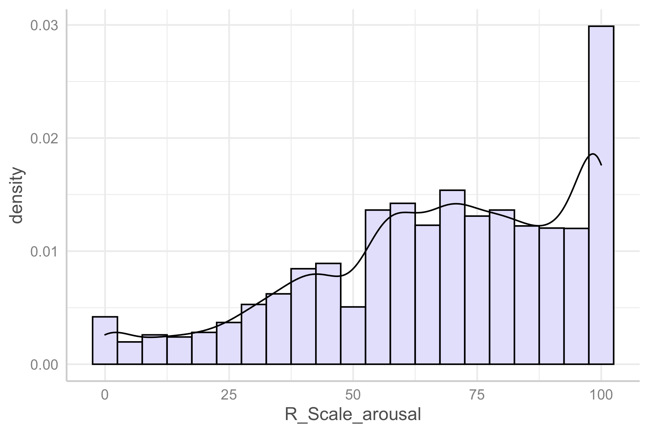

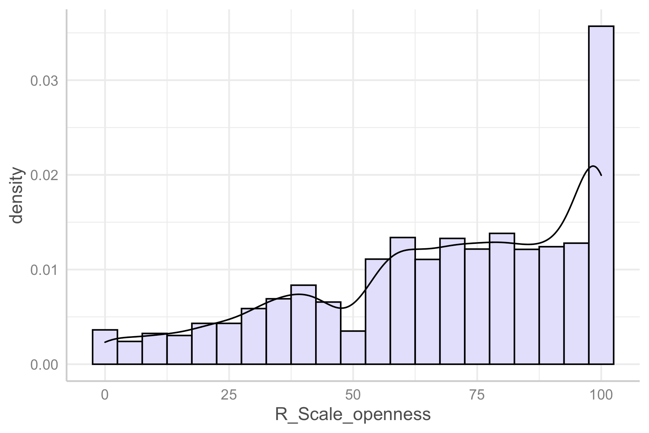

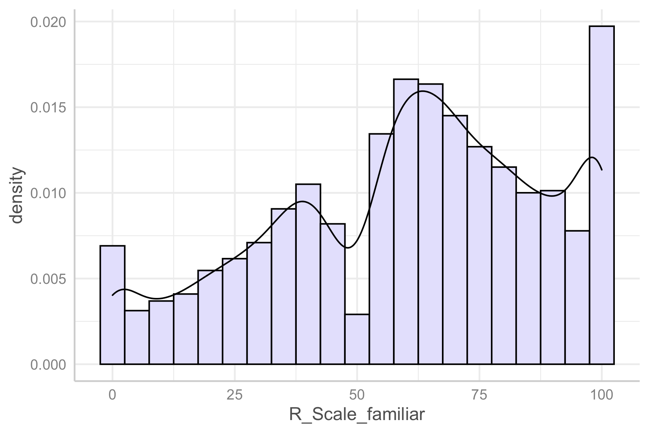

Supplement: Supplementary file 2 [file Data_Sheet_2.docx]
