# Supplementary material for: The other blue: Role of sky in the perception of nature
Source: Front Psychol. 2022 Oct 28;13:932507. doi: 10.3389/fpsyg.2022.932507 (PMC9651055; doi:10.3389/fpsyg.2022.932507)
Supplement: Supplementary file 3 [file Data_Sheet_3.docx]

**Supplement:** Extended data. Extended modelling.

Sky affects the judgement of naturalness:

Extending the baseline model for sky conditions (*Naturalness ~ Percentage of trees + weather conditions + (1|sub) + (1|image)*) showed its effect on increased naturalness ratings is more pronounced in high tree visibility and cloudy sky conditions and less so in the grey sky with low tree visibility. Model’s estimates showed all conditions had significant effects on naturalness ratings with variance explained by fixed effects expressed via marginal R^2^_trees_=.117 and conditional R^2^_trees_=.418. The model performed significantly better than baseline (X^2^(2)=8.95,p<.05). Therefore, the term was retained for further modelling with the addition of season as final term: *Naturalness ~ percentage of trees + weather conditions + season + (1|sub) + (1|image).* The addition of the seasonal effect (all estimates significant at p<.05) showed a substantial improvement in the model’s prediction of the naturalness ratings (likelihood ratio test showed improvement in performance over the previous model with X^2^(2)=11.26,p<.001). Higher tree visibility and winter conditions with a cloudy sky were all responsible for the summary effect on increased naturalness ratings. Meanwhile, blue skies dominated the effect not only in winter but also during spring/summer.
