# Supplementary material for: The other blue: Role of sky in the perception of nature
Source: Front Psychol. 2022 Oct 28;13:932507. doi: 10.3389/fpsyg.2022.932507 (PMC9651055; doi:10.3389/fpsyg.2022.932507)
Supplement: Supplementary file 4 [file Data_Sheet_4.docx]

**Supplement:** Extended data. GLMM Computation:

To test the hypotheses, we have modelled associations with a generalized linear mixed-effect model (GLMM) with a log link function. The log link function was selected to ensure positively fitted values. Initially, we have constructed a baseline model (DV ~ sum of random effect intercepts) based on Poisson distribution, typically used for data produced by similar experiments. However, model diagnostics detected significant overdispersion (Breslow & Clayton, 1993; Garson, 2013). Overdispersion occurs due to the observed variance being higher than the variance of a theoretical model. Since negative-binomial distributions are discrete distributions with a lower bound of zero (and zero-tolerant) just like Poisson distributions, we have selected the negative-binominal family GLMM for the baseline model and tested the difference in performance with the likelihood ratio test. The negative-binominal GLMM performed better than Poisson GLMM, hence we chose to proceed with the negative-binominal model family and log link function.

Model terms were tested with a likelihood ratio test and type III sum of squares. The goodness of fit was assessed with marginal and conditional R^2^ calculated using the method developed by Nakagawa and Schielzeth (2013). When necessary, models were stepwise compared with likelihood ratio test to baseline model and using Akaike Information Criterion (AIC) as a performance indicator.

Breslow, N. E., & Clayton, D. G. (1993). Approximate Inference in Generalized Linear Mixed Models. *Journal of the American Statistical Association, 88*(421), 9-25. doi:10.1080/01621459.1993.10594284

Nakagawa, S., & Schielzeth, H. (2013). A general and simple method for obtaining R2 from generalized linear mixed‐effects models. *Methods in ecology and evolution, 4*(2), 133-142.
