# Supplementary material for: The other blue: Role of sky in the perception of nature
Source: Front Psychol. 2022 Oct 28;13:932507. doi: 10.3389/fpsyg.2022.932507 (PMC9651055; doi:10.3389/fpsyg.2022.932507)
Supplement: Supplementary file 5 [file Data_Sheet_5.docx]

**Supplement**: List of covariates.

| SES_1_1 | Sex |
| --- | --- |
| SES_1_2 | Age |
| SES_1_3 | Nationality |
| SES_1_4 | Education |
| SES_1_5 | Years in education (Primary Education + Secondary Education + Further Education + Higher Education): How many years have you spent in education in total? |
| SES_1_6 | What is your average monthly income? |
| SES_1_7 | Profession |
| SES_1_8 | If Student: what do you study |
| SES_1_9 | If student: Which semester are you currently attending? |
| SES_1_10 | If professional: How experienced are you in years? |
| SES_1_11 | screen size in inches |
| SES_2_1 | Where did you grow-up? |
| SES_2_2 | What is your native language? |
| SES_2_3 | up to age of 15 how many years you lived in city/town/rural area |
| SES_2_4 | how many days up to age of 15 you spent in the countryside |
| SES_2_5 | How many squaremeters your growing house had |
| SES_2_6 | how many rooms growing house had |
| SES_3_1 | What type of an accommodation do you live in? |
| SES_3_2 | How many rooms does your accommodation have excluding hall, bathroom and kitchen? |
| SES_3_3 | In which room of your apartment / house do you spend most of the time? (If you spend most of your time in more than one room, please list two / more rooms.) |
| SES_3_4 | How many square meters is your living room / bedroom or the room in which you spend most of your time? (Please only enter whole numbers and no letters in the field.) |
| SES_3_5 | how many people you live with? |
| SES_3_6 | How much of the window view in the room you spend most of your time in is sky, green, and man-made? |
| SES_3_7 | How sunny is your apartment? |
| SES_3_8 | How many sun-hours do you have in the livingroom/bedroom/room in which you are spending most of your time? |
| SES_3_9 | How much nature (trees, lake, grass, flowers, bushes…) is there around your house? |
| SES_4_1 | What are the first three words you think of when you hear the word 'city'? |
| SES_4_2 | What are the first three words you think of when you hear the word 'nature'? |
| SES_4_3 | How important is time in nature to you? |
| SES_4_4 | Do you work from home? |
| SES_4_5 | If yes, how long have you been working from home(in months)? |
| SES_4_6 | How much time do you spend outdoors on an average day? |
| SES_4_7 | How often do you visit nature? |
| SES_4_8 | Where are you exactly doing this experiment (living room, study, cafe, library, bedroom etc.)? |
| SES_4_9 | Are you with company or alone at the moment? |
| ARA_1_1 | I visit museums or go to musical/dance performances. |
| ARA_1_2 | I notice beauty when I look at art. |
| ARA_1_3 | I am emotionally moved by music. |
| ARA_1_4 | When viewing artistic works, I am impressed by their harmony. |
| ARA_1_5 | I sculpt, paint, draw, direct films, or do design work. |
| ARA_1_6 | When I look at art, I feel positive energy or invigoration. |
| ARA_1_7 | I write poetry or fiction. |
| ARA_1_8 | When I look at art, my heart beats faster, I perspire, get dizzy, or have other physical effects. |
| ARA_1_9 | I appreciate the visual design of buildings. |
| ARA_1_10 | Presently (or in the past) I take (or have taken) classes in art,  creative writing, or aesthetics. |
| ARA_1_11 | I experience awe, fear, or a feeling of being overwhelmed when looking at art. |
| ARA_1_12 | When viewing artistic works, I feel a oneness, unity or connectedness with the universe/nature/existence/my deity. |
| ARA_1_13 | I am deeply moved when I see art. |
| ARA_1_14 | I experience joy, serenity, or other positive emotions when looking at art. |
| CNS_1_1 | I often feel a sense of oneness with the natural world around me. |
| CNS_1_2 | I think of the natural world as a community to which I belong. |
| CNS_1_3 | I recognize and appreciate the intelligence of other living organisms. |
| CNS_1_4 | I often feel disconnected from nature. |
| CNS_1_5 | When I think of my life, I imagine myself to be part of a larger cyclical process of living. |
| CNS_1_6 | I often feel a kinship with animals and plants. |
| CNS_1_7 | I feel as though I belong to the Earth as equally as it belongs to me. |
| CNS_1_8 | I have a deep understanding of how my actions affect the natural world. |
| CNS_1_9 | I often feel part of the web of life. |
| CNS_1_10 | I feel that all inhabitants of Earth, human, and nonhuman, share a common ‘life force’. |
| CNS_1_11 | Like a tree can be part of a forest, I feel embedded within the broader natural world. |
| CNS_1_12 | When I think of my place on Earth, I consider myself to be a top member of a hierarchy that exists in nature. |
| CNS_1_13 | I often feel like I am only a small part of the natural world around me, and that I am no more important than the grass on the ground or the birds in the trees. |
| CNS_1_14 | My personal welfare is independent of the welfare of the natural world. |
| STAI_1_1 | I feel calm |
| STAI_1_2 | I feel secure |
| STAI_1_3 | I feel tense |
| STAI_1_4 | I feel strained |
| STAI_1_5 | I feel at ease |
| STAI_1_6 | I feel upset |
| STAI_1_7 | I am presently worrying over possible misfortunes |
| STAI_1_8 | I feel satisfied |
| STAI_1_9 | I feel frightened |
| STAI_1_10 | I feel comfortable |
| STAI_1_11 | I feel self-confident |
| STAI_1_12 | I feel nervous |
| STAI_1_13 | I feel jittery |
| STAI_1_14 | I feel indecisive |
| STAI_1_15 | I feel relaxed |
| STAI_1_16 | I feel content |
| STAI_1_17 | I am worried |
| STAI_1_18 | I feel confused |
| STAI_1_19 | I feel steady |
| STAI_1_20 | I feel pleasant |
| STAI_2_1 | I feel pleasant |
| STAI_2_2 | I feel nervous and restless |
| STAI_2_3 | I feel satisfied with myself |
| STAI_2_4 | I wish I could be as happy as others seem to be |
| STAI_2_5 | I feel like a failure |
| STAI_2_6 | I feel rested |
| STAI_2_7 | I am “calm, cool and collected” |
| STAI_2_8 | I feel that difficulties are piling up so that I cannot overcome them |
| STAI_2_9 | I worry too much over something that really does not matter |
| STAI_2_10 | I am happy |
| STAI_2_11 | I have disturbing thoughts |
| STAI_2_12 | I lack self-confidence |
| STAI_2_13 | I feel secure |
| STAI_2_14 | I make decisions easily |
| STAI_2_15 | I feel inadequate |
| STAI_2_16 | I am content |
| STAI_2_17 | Some unimportant thought runs through my mind and bothers me |
| STAI_2_18 | I take disappointments so keenly that I can’t put them out of my mind |
| STAI_2_19 | I am a steady person |
| STAI_2_20 | I get in a state of tension or turmoil as I think over my recent concerns and interests |
| BIG-5_1_1 | is reserved |
| BIG-5_1_2 | is generally trusting |
| BIG-5_1_3 | tends to be lazy |
| BIG-5_1_4 | is relaxed, handles stress well |
| BIG-5_1_5 | has few artistic interests |
| BIG-5_1_6 | is outgoing, sociable |
| BIG-5_1_7 | tends to find fault with others |
| BIG-5_1_8 | does a thorough job |
| BIG-5_1_9 | gets nervous easily |
| BIG-5_1_10 | has an active imagination |
